# Protein assemblies in the *Arabidopsis thaliana* chloroplast compartment

**DOI:** 10.3389/fpls.2024.1380969

**Published:** 2024-08-16

**Authors:** Noah Ditz, Hans-Peter Braun, Holger Eubel

**Affiliations:** Department of Plant Proteomics, Institute of Plant Genetics, Leibniz Universität Hannover, Hannover, Germany

**Keywords:** photosynthesis, protein complexes, high light, low light, protein assembly, chloroplast transcription, protein:protein interactions, cross-linking

## Abstract

**Introduction:**

Equipped with a photosynthetic apparatus that uses the energy of solar radiation to fuel biosynthesis of organic compounds, chloroplasts are the metabolic factories of mature leaf cells. The first steps of energy conversion are catalyzed by a collection of protein complexes, which can dynamically interact with each other for optimizing metabolic efficiency under changing environmental conditions.

**Materials and methods:**

For a deeper insight into the organization of protein assemblies and their roles in chloroplast adaption to changing environmental conditions, an improved complexome profiling protocol employing a MS-cleavable cross-linker is used to stabilize labile protein assemblies during the organelle isolation procedure.

**Results and discussion:**

Changes in protein:protein interaction patterns of chloroplast proteins in response to four different light intensities are reported. High molecular mass assemblies of central chloroplast electron transfer chain components as well as the PSII repair machinery react to different light intensities. In addition, the chloroplast encoded RNA-polymerase complex was found to migrate at a molecular mass of ~8 MDa, well above its previously reported molecular mass. Complexome profiling data produced during the course of this study can be interrogated by interested readers via a web-based online resource (https://complexomemap.de/projectsinteraction-chloroplasts).

## Introduction

Chloroplast functions, such as light harvesting, biosynthesis of reduced organic compounds, gene expression, or protein import, are of fundamental importance for plant life. It is estimated that up to 3600 distinct nuclear encoded proteins are targeted to chloroplasts (Leister, 2003). In addition, the chloroplast genome contains 87 protein-encoding genes (Dobrogojski et al., 2020). Chloroplast sub-fractionation analysis assigned >1300 proteins to organelle sub-compartments, such as the two envelope membranes, the stroma, the thylakoid membranes, the thylakoid lumen, plastoglobuli, and nucleoids (Sun et al., 2009; Ferro et al., 2010). Within all these sub-compartments, well defined protein:protein interactions (PPIs) are reported (Peltier et al., 2006; Olinares et al., 2010; Suorsa et al., 2015; Takabayashi et al., 2017). PPIs are often unique in respect to their specificity, frequency and stability. Permanent and stable PPIs result in the formation of functional units in the form of protein complexes. In some cases, protein complexes associate to even larger structures, termed super- or mega-complexes [as is, for example, the case for the electron transfer chain components in chloroplasts and mitochondria (Krause et al., 2004; Rantala et al., 2017)]. Protein complexes and their higher-level assemblies usually appear well defined in terms of protein composition and stoichiometry. However, the *in vivo* situation may be more complex than the current biochemical data suggest. The use of detergents inevitably destabilizes (some) PPIs. As a result of this, only stable ‘degradation products’ of higher molecular mass assemblies are observed. The PPI landscape of chloroplasts is also shaped by a wealth of transient PPIs.

When compared to randomly distributed proteins, PPIs serve several beneficial aims. They reduce the water accessible surface area, which is favorable in energetic terms and increases protein solubility in cellular environments experiencing molecular crowding. Furthermore, they boost metabolic efficiency by bringing the reaction centers of two enzymes catalyzing consecutive steps of a pathway into closer proximity (Pollmann et al., 2019). Protein assemblies can also create microenvironments, in which labile substrates are stabilized, substrate concentrations are increased, and pH-values are adjusted to better meet the requirements of the enzymes involved (Sweetlove and Fernie, 2013, 2018). Additionally, protein complexes and microdomains may help in controlling metabolic interactions between different (possibly competing) pathways in order to adjust metabolism to changing conditions (Ruiz-Sola et al., 2016; Le et al., 2022). However, while experimental evidence for such microdomains is scarce, it is accepted that a short-term adjustment of metabolism (for example during quickly changing light conditions) by production and degradation of respective enzymes is wasteful, and that further levels of regulation, such as post-translational modifications (PTMs), exist. Some of these PTMs may also influence dynamic PPIs and therefore indirectly serve in the regulation of metabolic processes.

Within the chloroplast compartment, high molecular mass protein complexes organize gene expression, photosynthesis, and protein import, among others. A fine example for the diverse functions and dynamics of chloroplast protein complexes is photosystem (PS) II. Together with varying numbers of its associated light harvesting complexes (LHCII), it is densely packed into stacked thylakoid membranes (grana), taking up 70–80% of the membrane area (Haferkamp et al., 2010; Rantala et al., 2020). PSII-LHCII super-complexes form semi-crystalline arrays within the plane of a thylakoid membrane, that also interact with adjacent grana membranes to increase grana stacking and to facilitate efficient light harvesting under low light conditions (Kouřil et al., 2013; Albanese et al., 2016, 2020). Under high light intensities, these PSII structures disassemble, and the grana margin surface area increases (Kirchhoff, 2014). Structural rearrangements of grana also include the decrease of grana disc diameter and thylakoid unstacking (Khatoon et al., 2009; Sujith et al., 2014), thus reducing the absorption of excess radiation (Grinzato et al., 2020) and allowing access of the bulky thylakoid FTSH protease complex to the D1 subunit of PSII for degradation and subsequent replacement (Theis and Schroda, 2016). In this repair cycle, PSII super-complexes disassemble, to enable migration of damaged PSII monomers to grana margins and stroma lamellae, where all components of the PSII repair cycle form a micro-environment for efficient repair (Theis and Schroda, 2016).

Complexome profiling (CP) is used to investigate protein assemblies in an unbiased and untargeted manner. While most methods examining PPIs are targeted approaches that rely on genetic strategies, such as co-immunoprecipitation, yeast-two-hybrid or bimolecular fluorescence complementation, CP is a biochemical method that can be applied to any tissue, cell type or organelle without the need of genetic transformation (Arnold and Braun, 2022). It is suited to identify unknown subunits of known complexes (Heide et al., 2012; Vukotic et al., 2017; Evers et al., 2021), to study protein complex assembly (Vidoni et al., 2017; Ligas et al., 2019; Lobo-Jarne et al., 2020; Röhricht et al., 2021), to identify high to medium abundant protein assemblies (Rugen et al., 2019), to investigate diseases (Sánchez-Caballero et al., 2016; Chatzispyrou et al., 2018; Gardeitchik et al., 2018; Alston et al., 2020), and to assess molecular masses (Rugen et al., 2019; Schröder et al., 2022a) and stoichiometries (Schwenk et al., 2012; Turecek et al., 2014) of protein complexes. The CP workflow is based on the mild solubilization of protein complexes, followed by their native separation in blue-native (BN) gels (Wittig and Malacarne, 2021). The gel lane is then cut into dozens of fractions, each of which covering a small part of the total mass range of the gel. Shotgun proteome analysis of each individual fraction is then performed, the results of which are used to produce abundance profiles for all identified protein groups over the gel’s mass range. Finally, hierarchical clustering of these protein abundance profiles reveals proteins with similar migration profiles. Proteins sharing abundance peaks at positions higher than those of the singular proteins are likely to interact with each other by stable PPIs. Depending on the complexity of the sample, the interactions of thousands of proteins can be assessed in a single experiment (van Strien et al., 2021; Wittig and Malacarne, 2021; Arnold and Braun, 2022; Schröder et al., 2022a, b). However, CP does not come without technical limitations. Native gel systems are limited in respect to the maximum upper molecular mass of protein complexes to enter the gel matrix. It also bears the risk of destabilizing labile PPIs upon solubilization or the subsequent mechanical strain during electrophoretic separation. Moreover, CP suffers from co-migration of proteins, which belong to separate protein complexes of similar molecular masses, and are thus often misinterpreted as interacting with each other in a single complex. These technical limitations can be attenuated by combining cross-linking coupled mass spectrometry (XL-MS) and CP into a single workflow. Use of the MS-cleavable cross-linker (XL) disuccinimidyl dibutyric urea (DSBU) stabilizes labile PPIs during solubilization and electrophoresis. Furthermore, it allows identification of proteins located within close proximity of each other directly by MS.

In total, 17 complexome maps integrating 813 individual MS runs covering 4077 unique protein groups are presented here, which can be explored in detail on our complexome profiling portal at https://complexomemap.de/projects-interaction-chloroplasts/

For access to the underlying MS-data, please visit http://massive.ucsd.edu/ProteoSAFe/status.jsp?task=b140b78d63a64fd7a14b3a80a5cff2bc.

## Results and discussion

### Mass calibration of lpBN gels using mitochondrial protein complexes and super-complexes and assessment of contamination in the chloroplast isolate

**Figure 1:**
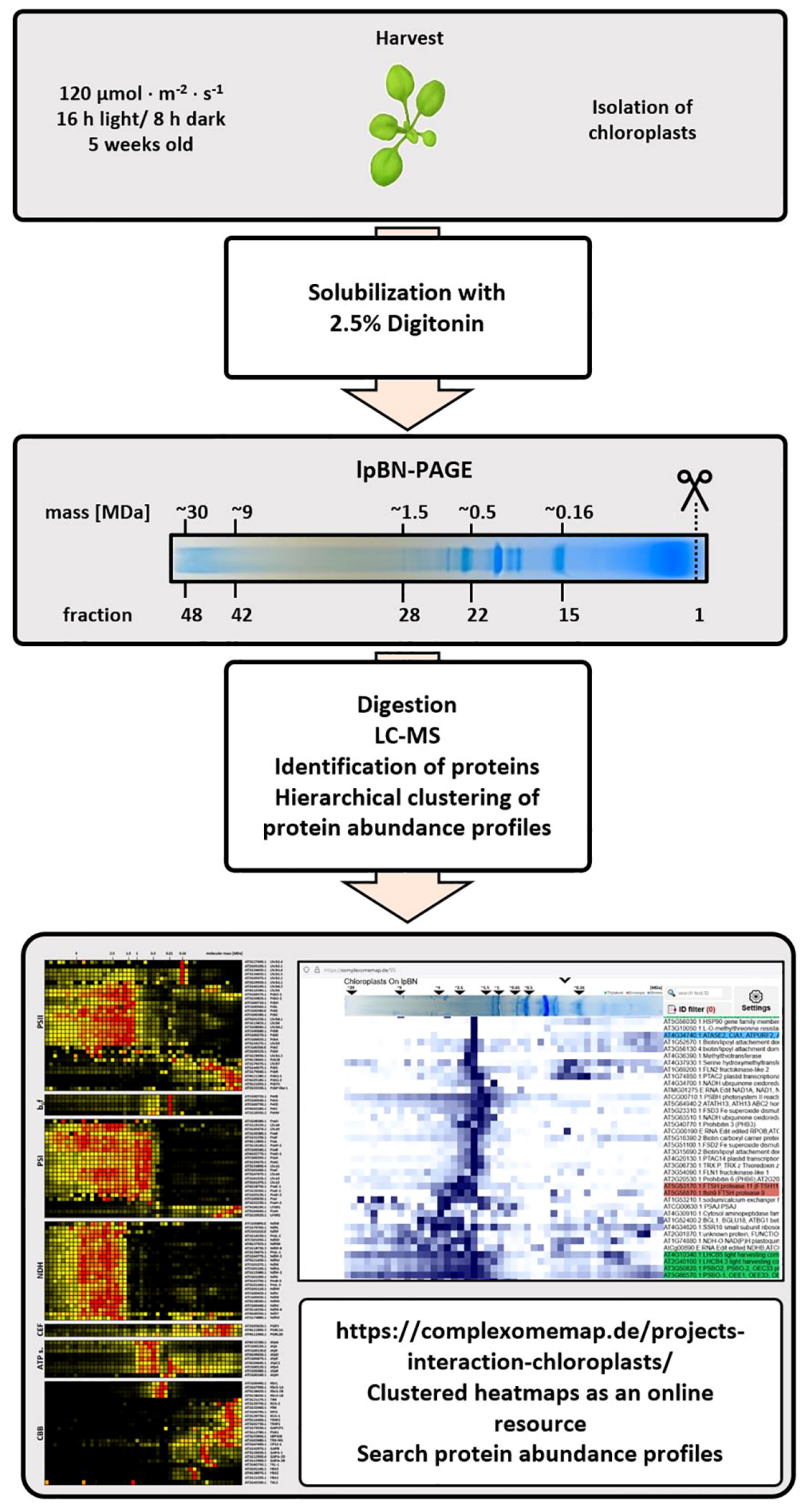
Flowchart of the complexome profiling workflow. *Arabidopsis thaliana* plants (ecotype Columbia-0) were grown under long day conditions (16 h light, 8 h darkness) using a photon flux density (PFD) of 120 µmol ∙ m^-2^ ∙ s^-1^ (first panel). Chloroplasts and mitochondria were isolated in parallel. Chlorophyll and protein concentrations were adjusted as explained in the experimental procedures before solubilization with 2.5% [w/v] digitonin (second panel). Solubilized protein complexes were separated according to their apparent molecular mass on the same lpBN-PAGE and stained gel lanes were sliced into 48 fractions, each with a height of 4 mm, a width of 10 mm and a thickness of 1.5 mm (third panel). Each fraction was subjected to tryptic in-gel digestion before peptide mixtures of each fraction were analyzed by LC-timsTOF-MS (fourth panel). Recorded spectra were analyzed by MaxQuant (Cox and Mann, 2008) to generate abundance profiles, which were then submitted to hierarchical clustering using the NOVA software (Giese et al., 2015, fifth panel). Clusters of protein complex subunits were extracted and, where necessary, curated manually by adding known subunits which were not part of the cluster. Access to complete heatmaps containing thousands of protein abundance profiles is found at https://complexomemap.de/projects-interaction-chloroplasts/ (bottom panel).

**Figure 2:**
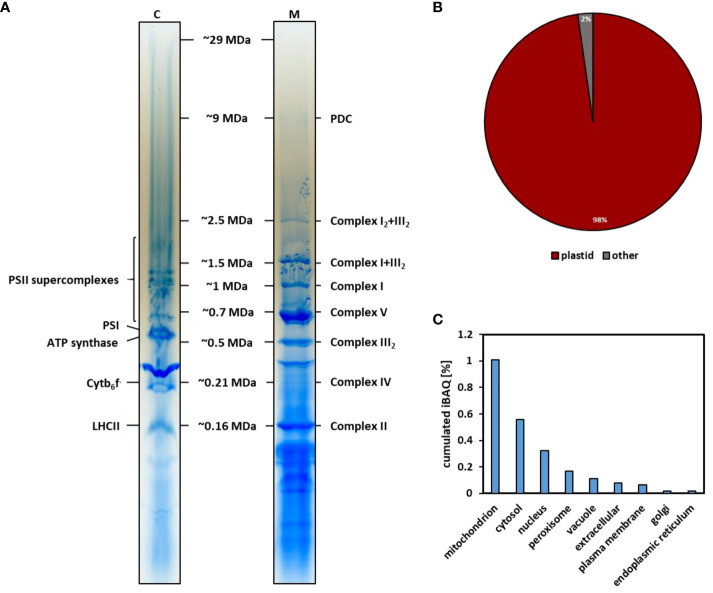
Separation of chloroplast and mitochondrial proteins by lpBN-PAGE for mass calibration and purity assessment of organellar fractions. **(A)** lpBN-PAGE of chloroplast (C) and mitochondrial (M) protein complexes. 125 µg of mitochondrial protein and chloroplast protein equivalent to 20 µg chlorophyll were solubilized with 2.5% [w/v] digitonin and separated in the same lpBN-gel. Resulting gel lanes were Coomassie-stained and cut into 48 fractions. Molecular masses of known mitochondrial respiratory chain protein complexes and super-complexes (I, II, III_2_, IV, V, I+III_2_, I_2_+III_2_) as well as PDC and OGDC were used for mass calibration of gel fractions. Molecular masses expressed in MDa are shown in between the gel lanes. **(B)** Proportion of plastid protein abundance in the chloroplast complexome. Each fraction was subjected to tryptic in-gel digestion followed by UHPLC-separation coupled to timsTOF-MS. Subcellular localization of proteins identified across all fractions was predicted using SUBAcon (Hooper et al., 2014). In conjunction with cumulated iBAQ values for each protein, this was used to calculate the contribution of cellular compartments to each isolate. **(C)** Proportion of protein abundance in the chloroplast isolate assigned to other cell compartments by SUBAcon.

The lpBN gels used in the CP workflow described here allow separation of PPIs up to 30 MDa (Strecker et al., 2010; Rugen et al., 2021) (**Figure 1**). Since this separation method differs from the native gels previously used to separate chloroplast protein complexes, a mass-calibration of CP fractions was performed. The molecular masses of plant mitochondrial complexes and super-complexes are well defined from previous studies using lpBN-PAGE (Rugen et al., 2019, 2021). In addition, recent cryogenic electron microscopy (cryo-EM) studies on respiratory complex I and its I+III_2_ super-complex (Klusch et al., 2021, 2022) provide precise masses for these mitochondrial electron transfer chain components. As such, mitochondrial protein complexes and super-complexes lend themselves for a mass-calibration of lpBN-separated chloroplast protein assemblies. To this end, chloroplasts and mitochondria were isolated in parallel from five-week-old *Arabidopsis* rosette leaves. Chloroplast protein equivalent to 20 µg chlorophyll and 125 µg protein of the mitochondrial fraction were separately solubilized in 2.5% [w/v] digitonin and subsequently separated on different lanes of the same lpBN-gel (**Figure 2A**). After Coomassie staining, resulting gel lanes were cut into gel-slices from bottom to top (width, ~10 mm; height, ~4 mm), before being subjected to tryptic in-gel digestion. The peptide content of each fraction was separated by reverse-phase (RP) ultra-high performance liquid chromatography (UHPLC) and analyzed by trapped ion mobility spectrometry time of flight (timsTOF) mass spectrometry. On average, 1113 protein groups were identified in a single gel fraction of the chloroplast complexome (resulting in 1576 unique protein groups across all fractions), whereas 1073 protein groups were detected on average in mitochondrial complexome fractions (resulting in 1639 unique protein groups across all fractions). For both organelles, the number of identified protein groups is highest in low molecular mass gel fractions and slowly drops off towards the high molecular mass fractions (**Supplementary Figure 1**). Abundance of individual proteins in lpBN fractions was estimated by intensity based absolute quantification (iBAQ) (Schwanhäusser et al., 2011) values. Protein groups were assigned to a cellular compartment using the SUBAcon algorithm (Hooper et al., 2014). After iBAQ-values for each protein group in each gel fraction were summed up for each compartment, their relative share in the chloroplast isolate could be calculated (**Figures 2B, C**). Ninety-eight percent of the protein abundance in the chloroplast fraction is of chloroplast origin, indicating a high level of homogeneity within the organelle isolate. Among the protein groups from co-purifying organelles, mitochondrial protein groups rank highest, followed by cytosolic and nuclear protein groups.

**Figure 3:**
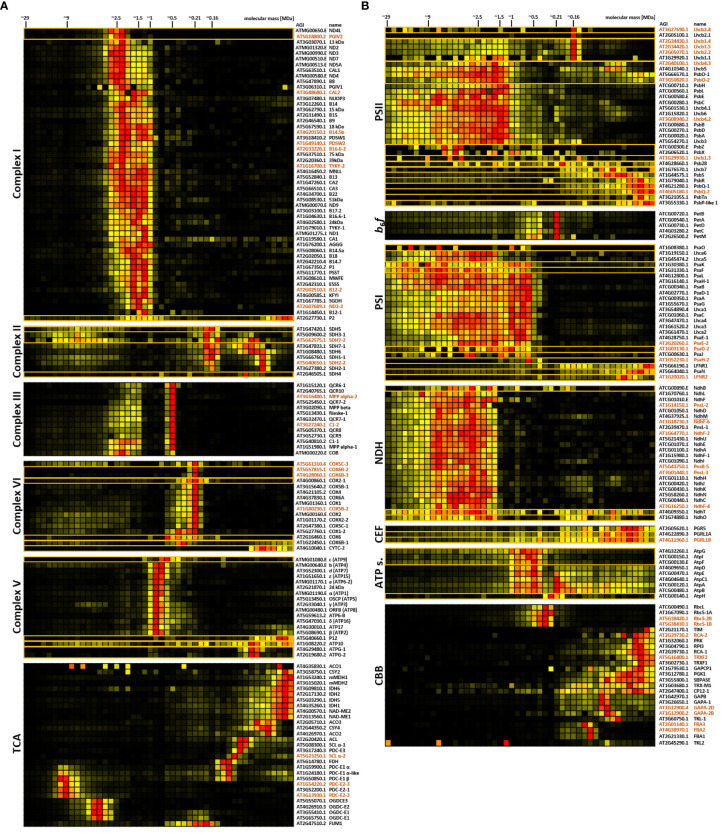
Protein abundance profiles of major protein complex subunits in mitochondria **(A)** and chloroplasts **(B)**. Protein abundance profiles (horizontal rows) as deduced from the complexome profiling workflow show normalized iBAQ values across the 48 fractions for each identified protein. Individual clusters are extracted from the original heatmaps (**Supplementary Figures 2**, **3**). In some cases, known complex subunits, which were absent from the clusters were manually added (indicated by orange boxes. More than one abundance profile within a single box also form a cluster in the complete complexome map). Also, obviously co-migrating proteins were removed manually. Heatmaps of the Tricarboxylic acid (TCA) - and Calvin-Benson-Bassham cycle (CBB) cycle were largely produced by manual curation, since interactions between enzymatic units of the TCA cycle are not observed under the conditions applied here. Highest protein abundance is depicted in red, low abundance in yellow, and non-detectable abundance is displayed in black. Molecular masses of fractions are indicated at the top. Arabidopsis gene identifiers (AGIs) and protein names are found to the right of the heatmaps (isoforms are marked in brown letters), while corresponding complexes and pathways are indicated to the left. Mitochondrial complexome maps focus on respiratory complexes I-V. In adddition, TCA enzymes, the pyruvate dehydrogenase complex (PDC), and malic enzyme (ME) are shown together with TCA-cycle components. For chloroplasts, abundance profiles of proteins involved in photosystem II (PSII), cytochrome *b*_6_ *f*-complex (*b*_6_ *f*), photosystem I (PSI), chloroplast NDH complex (NDH), antimycin-A-sensitive cyclic electron flow (CEF), ATP-synthase (ATP s.), and CBB are shown.

iBAQ values were also used to produce abundance profiles for each protein group along the mitochondrial gel lane. Subunits of respiratory protein complexes I-V (CI-CV), their super-complexes, as well as the pyruvate dehydrogenase complex (PDC) and the 2-oxoglutarate dehydrogenase complex (OGDC), migrate closely to their previously reported positions (Senkler et al., 2017; Rugen et al., 2021). Hierarchical clustering results in well-defined clusters for known mitochondrial complexes and super-complexes, that require only minimal manual curation in order to add known, but missing subunits (**Figure 3A**; **Supplementary Figure 2**). Abundance peaks of CI are in the same fractions as in previous lpBN-PAGE CP experiments (Rugen et al., 2019; Rugen et al., 2021). Due to accurate molecular mass data obtained from high-resolution cryo-EM studies, their peak masses are defined as 1.0 MDa for the singular complex (Klusch et al., 2021), 1.5 MDa for the I+III_2_ super-complex (Klusch et al., 2022), and 2.5 MDa for the I_2_+III_2_ super-complex. CIII is most abundant in its dimeric form (III_2_; 0.5 MDa), but also shares peaks with CI subunits at 1.5 MDa and 2.5 MDa as described above. Subunits of CII and CIV form sharp clusters at previously defined masses of 0.16 MDa and 0.21 MDa (Senkler et al., 2017; Rugen et al., 2021), respectively (**Figure 3A**).

Abundance in other areas either represents breakdown products, assembly intermediates, or participation in other super-complexes. A sharp abundance peak is also present for CV (mass definition 700 kDa), but moderate to low abundance is also detected in higher fractions, probably representing dimeric and multimeric ATP synthase complexes, which form rows at the curved edges of cristae membranes (Davies et al., 2012). Subunits of PDC and OGDC form two distinct clusters with peaking protein abundance defined as ~9 MDa and ~4 MDa, respectively (Rugen et al., 2019, 2021; **Figure 3A**). Fitment of these masses onto the calibration curve is excellent (**Supplementary Figure 4**), resulting in a R^2^ value of 0.9946, thus showcasing the suitability of mitochondrial protein complexes and super-complexes as high molecular mass markers.

### PSII complexes associate in a large protein continuum upon solubilization with digitonin

With the notable exceptions of the cytochrome-*b*_6_ *f*-complex (*b*_6_ *f*) and some stromal enzymes involved in the Calvin-Benson-Bassham (CBB) cycle, abundance peaks of photosynthesis-related protein groups in the chloroplast complexome profile are considerably broader than the peaks in the mitochondrial complexome (**Figure 3B**). Especially subunits of PSII, PSI, and the chloroplast NDH complex (which is homologous to the mitochondrial NADH dehydrogenase complex and also termed ‘chloroplast complex I’) are distributed across a wide mass range (**Figure 3B**). Depending on solubilization-buffer composition and detergent concentration, digitonin treatment of chloroplast fractions was previously reported to allow only partial solubilization of the core membrane stacks of thylakoid grana (Järvi et al., 2011; Suorsa et al., 2015). The slightly harsher detergent dodecyl-β-D-maltoside (DDM) was found to be more efficient in this respect, albeit at the expense of higher dissociation rates of PSII super-complexes (Järvi et al., 2011; Rantala et al., 2017). The best compromise between solubilization success of thylakoid protein complexes and avoidance of super-complex disruptions is achieved by dissolving digitonin in aminocaproic-acid buffer (Rantala et al., 2017), which is similar to the strategy applied in our CP workflow. Since PSI is exclusively present in unstacked thylakoid membranes or at grana margins, it is easier accessible to detergents than PSII complexes, which are predominantly located in stacked thylakoid grana. Efficient PSII solubilization thus requires the use of harsher detergents (Rantala et al., 2017). Since PSI is easier to solubilize than PSII, the stoichiometry between these two complexes should be an adequate proxy for efficient solubilization of thylakoid membranes. Here, the cumulated abundance ratio of PSII over PSI subunits is 1.8 (**Supplementary Table 1**), closely matching the previously reported value of 1.7 (McKenzie et al., 2020), thus indicating that solubilization itself is most likely not responsible for the broad distribution of PS complexes in lpBN gels. Growing evidence suggests that PSII complexes are a hub of thylakoid protein interactions. They form semi-crystalline arrays within the thylakoid membrane plane (Kirchhoff et al., 2007; Kouřil et al., 2013), and also interact with PSII-assemblies in adjacent grana membranes (Albanese et al., 2017). It is also suggested that a ‘lake’ of free LHCII trimers connects the entire photosynthetic machinery (Grieco et al., 2015). Both effects may contribute to the peak broadening observed here. Our data show that Lhcb4, Lhcb5 and Lhcb6, which link LHCII to PSII (Boekema et al., 1999), share abundance peaks with subunits of the PSII complex (**Figure 3B**). In contrast, proteoforms of Lhcb1, Lhcb2 and Lhcb3 show their highest abundance at 0.16 MDa (**Figure 3B**), which is the mass of free trimeric LHCII (Järvi et al., 2011; Espinas et al., 2016), indeed supporting the notion that large amounts of PSII trimers are not tightly bound to PSII.

### PSI forms high molecular mass assemblies that may include other thylakoid protein complexes

PSI subunits are as broadly distributed as their PSII counterparts, but form a cluster that is clearly separated from other chloroplast complexes (**Figure 3B**; **Supplementary Figure 3**). The broad pattern may in part be due to PSI interacting with the NDH complex in different stoichiometries, ranging from one to five PSI copies associated with each NDH complex, as reported previously (Otani et al., 2018). There is also accumulating evidence for interactions between PSII and PSI at the grana margins (Järvi et al., 2011; Suorsa et al., 2015; Yokono et al., 2015, 2019; Rantala et al., 2017), which may also contribute to the observed shapes of PSI and PSII clusters.

### The majority of PGR5 and PGRL1 peak in low molecular mass fractions

All components of the antimycin A (AA) sensitive cyclic electron flow (CEF) pathway (PGR5, PGRL1A, and PGRL1B) peak at approximately 30 kDa (**Figure 3B**). While this fits to molecular masses reported for PGRL1A and PGRL1B, the molecular mass of PGR5 is considerably lower with only 14 kDa (Hertle et al., 2013). PGRL1 proteins form homo-dimers, but also hetero-dimers with PGR5 (Hertle et al., 2013). Due to the wide mass range observed here, it is not possible to make any statements on the product of hetero- and homo-dimerization events among these proteins. Interestingly, low to moderate (normalized) protein abundance is also detectable in high molecular mass fractions for all three proteins (**Figure 3B**). Co-immunoprecipitation experiments with PGRL1 previously showed interactions with the *b_6_f* complex (Hertle et al., 2013). In cyanobacteria, PGRL1 forms a CEF super-complex (Steinbeck et al., 2018) together with *b_6_f*, PSI and the ferredoxin-NADPH-reductase (FNR), although the existence of such a complex is disputed in plants (Johnson, 2011). However, the abundance profiles in the chloroplast complexome map suggest that components of the Arabidopsis AA-sensitive CEF migrate at molecular masses that would match the expected mass of such a super-complex.

### The chloroplast ATP synthase complex is fragile in the presence of digitonin

Abundance profiles of the chloroplast ATP synthase subunits peak at 0.48 MDa (**Figure 3B**). Another peak of high abundance is at 0.78 MDa (**Figure 3B**), which is close to the 0.72 MDa reported previously (Suorsa et al., 2015), and also matches that of the intact mitochondrial ATP synthase. Additionally, subunits of the F_1_ head of the complex peak at 0.26 MDa (**Figure 3B**). None of these peaks seem to match with that of the AtpH subunit (encoding the c-subunit forming the rotor sub-complex), which forms a peak at 0.3 MDa. With a mass of ~8 kDa, the 14-mer rotor assembly should have a mass of only ~112 kDa, therefore considerably less than the apparent molecular mass shown here. In any case, detachment from the ATP-synthase complexes suggests that the F_1_-part of the chloroplast ATP-synthase complex is destabilized in the presence of digitonin. This effect seems to be specific for the chloroplast ATP synthase complex, since its mitochondrial counterpart remains stable under the same conditions.

### Enzymes of the Calvin-Benson-Bassham cycle show little interactions among themselves or with other proteins

In contrast to the subunits of photosynthetic electron transport complexes and the ATP-synthase complex, the enzymes of the CBB cycle mostly form clusters at lower molecular masses (**Figure 3B**). An obvious exception to this are the RubisCO subunits, peaking between 0.35 MDa and 0.41 MDa (**Figure 3B**), which is comparable to prior reports of 0.43 MDa (Kügler et al., 1997; Behrens et al., 2013). Other notable exceptions are transketolase 2 (TKL2, at ~1 MDa), CBB cycle protein 12–1 (CP12–1), and TRX-M1. Among these enzymes of the CBB, only TKL is reported to co-localize with phosphoriboisomerase (PRI) and glyceraldehyde-3-phosphate dehydrogenase (GAPA) using nearest neighbor analysis (Anderson et al., 2006). Here, these enzymes display only weak co-migration behavior in low molecular mass fractions up to 0.26 MDa (**Figure 3B**).

In summary, individual chloroplast protein complexes migrate at similar molecular masses in lpBN gels and classical BN gels. Particularly the electron transfer complexes PSII, PSI, and the NDH complex show strong abundance in a molecular mass range between 0.5 MDa and 30 MDa, indicative of their participation in super-complexes or microdomains. In contrast to the situation in mitochondria, these interactions seem less well defined. However, based on the following considerations, we hypothesize that the broad distributions shown here actually reflect the native interaction patterns, rather than artefacts.

1.) Compared to classical BN-PAGE, CP also shows the protein content in-between visible bands. ‘Smearing’ may therefore also be present in classical chloroplast BN-based analyses, but is not detected when gels are examined with a focus on visible, discrete, Coomassie-stained gel bands.
2.) A considerable part of the ‘smear’ is located in the mass range between 5 MDa and 29 MDa. Most BN-based studies employ gels, in which the highest separated mass is considerably below the molecular mass at which ‘smearing’ occurs here.
3.) This ‘smear’ is only present in the chloroplast sample, whereas the mitochondrial reference sample shows much more focused complexes (**Figure 3**). It therefore cannot be caused by the gel system itself.
4.) Within the chloroplast sample, some protein complexes migrate in a well-defined manner (such as RubisCO, the chloroplast encoded RNA-polymerase complex, or the *b*_6_ *f* complex, **Supplementary Figure 3**). Therefore, smearing of the chloroplast electron transfer chain (ETC) complex subunits is not necessarily related to an artificial breakdown of the protein assemblies during organelle isolation, or the product of inefficient solubilization, but rather is a reflection of their true biological properties.

If chloroplast ETC subunits indeed migrate at these positions due to biological reasons, the sheer size of the assemblies suggests interactions between the individual ETC components. Such interactions were already reported previously by the use of BN-gel derivates (albeit not reaching the same maximum molecular mass limit achieved here; Iwai et al., 2010; Järvi et al., 2011; Grieco et al., 2015; Yokono et al., 2015; Rantala et al., 2017; Yokono et al., 2019). We conclude that the chloroplast ETC complexes tend to form large megacomplexes in the molecular mass range of 5 MDa to 30 MDa. *In vivo*, these super- and megacomplexes might be even larger, because it cannot be excluded that chloroplast protein assemblies degrade during organelle isolation, sample preparation, and electrophoresis. To further investigate this issue, a treatment with the membrane-permeable cross-linker DSBU after organelle isolation was introduced into the complexome profiling workflow (**Supplementary Figure 5**) in a bid to stabilize fragile PPIs. Being MS-cleavable, the use of this cross-linker also allows to identify the reaction sides of the cross linkers, thereby indicating which proteins reside in close proximity to each other within the chloroplast compartment. As such, the cross-linking data add further depth to CP cluster analysis. However, the use of cross-linkers necessitates adjustments to the CP workflow.

### Cross-linking affects solubilization and migration properties of chloroplast protein complexes

Freshly isolated chloroplasts were initially treated with 5 mM DSBU for 2 h before solubilization with 2.5% digitonin (1^st^ DSBU, 2^nd^ Dig) and separation via lpBN-PAGE (**Supplementary Figure 6A**). The resulting gel lane is devoid of any visible bands. MS analysis of the corresponding gel lane fractions confirmed a severe lack of protein abundance (**Supplementary Figure 6A**). In contrast, chloroplasts solubilized with digitonin before addition of DSBU (1^st^ Dig, 2^nd^ DSBU) behave similar to chloroplasts solubilized with digitonin in the absence of cross-linker (Dig) (**Supplementary Figure 6A**). DSBU cross-linking thus hinders the solubilization of protein complexes with digitonin. Experiments were therefore repeated with the slightly harsher detergent DDM. The resulting gel lane appears also nearly devoid of signal, but some faint bands are discernable (**Supplementary Figure 6B**). Indeed, MS-analysis of lpBN gel fractions revealed low protein abundance across all lpBN-PAGE fractions (**Supplementary Figure 6B**). The observed increase in protein abundance towards low molecular mass fractions in the samples treated with detergent before the cross-linker (“1^st^ DDM 2^nd^ DSBU” sample) when compared to its digitonin-solubilized counterpart, supports the results of previous studies reporting increased electrophoretic mobility of thylakoid protein complexes after DDM treatments (Järvi et al., 2011; Rantala et al., 2017). Low protein abundance across all lpBN-PAGE fractions in the 1^st^ DSBU 2^nd^ DDM sample indicates that DSBU can stabilize high molecular mass PPIs, even in the presence of harsher detergents. However, even DDM is only capable of solubilizing a small portion of these complexes (**Supplementary Figures 6B**, **7**). It is currently unclear, why DSBU-cross-linked chloroplasts show an increased resistance towards detergent solubilization. We speculate that this behavior is linked in some way to the high protein content in chloroplasts membranes (Haferkamp et al., 2010). Chloroplast membrane PPIs also exert a strong influence on thylakoid architecture, such as grana stacking and membrane curvature (Ryrif et al., 2005; Armbruster et al., 2013; Albanese et al., 2020). Stabilization of these structures by cross-linking may therefore also hinder solubilization. However, reaction efficiency of commercially available MS-cleavable cross-linkers is typically low, with estimates ranging between 1–5% (Steigenberger et al., 2020). Investigations to further unravel this effect are clearly necessary, but beyond the scope of this study.

### Potent detergents solubilize DSBU cross-linked chloroplast protein complexes more efficiently

**Figure 4:**
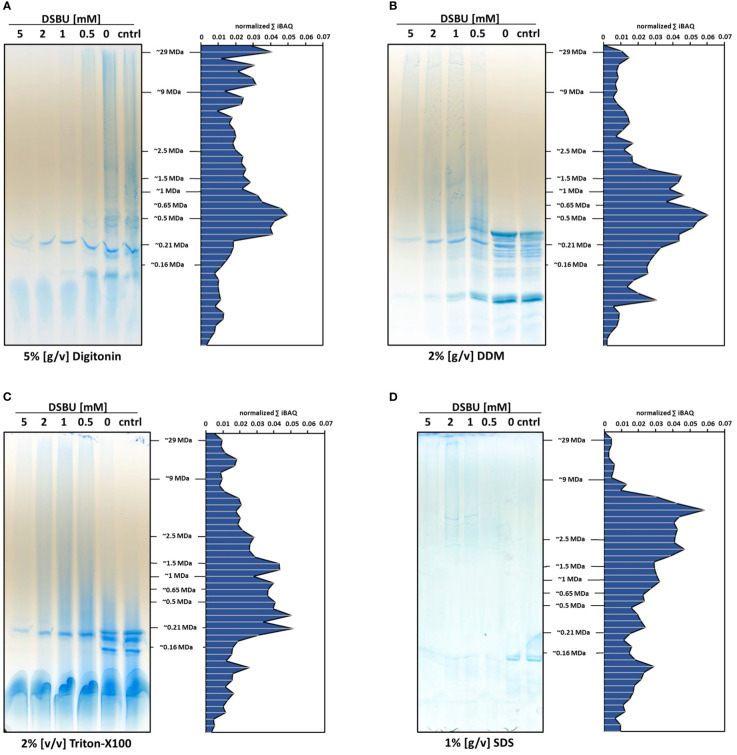
Influence of detergents on protein solubilization and distribution of DSBU cross-linked chloroplast proteins across lpBN-PAGE fractions. Left panels, freshly isolated chloroplasts were cross-linked with 0.5 - 5 mM DSBU for 2 h before solubilization with **(A)** 5% [w/v] digitonin; **(B)** 2% [w/v] dodecyl-b-D-maltoside (DDM); **(C)** 2% [v/v] Triton X-100; and **(D)** 1% [w/v] sodium dodecyl sulfate (SDS). After solubilization, protein equivalents to 20 μg chlorophyll were separated by lpBN-PAGE. Right panels, gel lanes obtained by using 1 mM DSBU were subjected to complexome profiling. Cumulating iBAQ values for all proteins within single fractions are displayed on the y-axes, molecular mass (in MDa) is indicated on the x-axes.

In a bid to improve the solubilization efficiency of DSBU-cross-linked chloroplast proteins, two additional detergents, Triton X-100 and sodium dodecylsulfate (SDS), were tested alongside digitonin and DDM. For this, increasing DSBU concentrations were combined with static detergent concentrations (**Figure 4**). In line with previous results, digitonin is not able to efficiently solubilize DSBU-cross-linked chloroplasts, resulting in a lack of protein abundance correlating with increasing cross-linker concentrations. Similar effects can be observed for DDM and Triton X-100, albeit to a lesser extent. Protein abundance of SDS-solubilized samples is seemingly lower than in the digitonin-, DDM-, and Triton X-100-solubilized samples on lpBN gels, but overall MS signal intensity is well within the range observed for the other detergents. In contrast to these, SDS does not require Coomassie being added to the sample before the gel run, which may lead to bands of lesser intensity, since the Coomassie colloids used for post-electrophoretic staining only attach to the protein moieties accessible at the gel surface, whereas Coomassie added before the gel run will bind to each and every protein molecule, thereby potentially increasing staining intensity. Interestingly, SDS-solubilization is the only treatment showing defined bands in the high molecular mass range upon cross-linking with 1 mM and 2 mM DSBU (**Figure 4**).

Focusing on the 1 mM DSBU treatment in **Figure 4** we conclude: Using 2% DDM, protein abundance is high in the lower to medium mass range, but declines considerably in fractions above 2 MDa. With 2% Triton X-100, maximum protein abundance is slightly lower compared to samples solubilized with 2% DDM, but the slope by which protein abundance decreases towards the maximum molecular mass is not as steep as with DDM. Interestingly, solubilization with 1% SDS produces a clear abundance shift towards high molecular masses, peaking in fractions of approximately 7.5 MDa (**Figure 4**). Between 2 and 9 MDa, protein abundance after SDS treatment is higher compared to any other detergent treatment tested here, going along with the highest number of identified DSBU intralinks and interlinks in these fractions (**Supplementary Figure 7**). Low SDS concentrations are thus able to efficiently solubilize high molecular mass chloroplast protein complexes stabilized by DSBU cross-linking. Since the use of SDS and the concomitant omission of Coomassie also guarantee the lowest level of unspecific association of proteins, it was decided to use this detergent for the following analyses.

### Protein abundance of the chloroplast encoded RNA polymerase shifts towards higher molecular masses upon DSBU cross-linking and SDS solubilization

**Figure 5:**
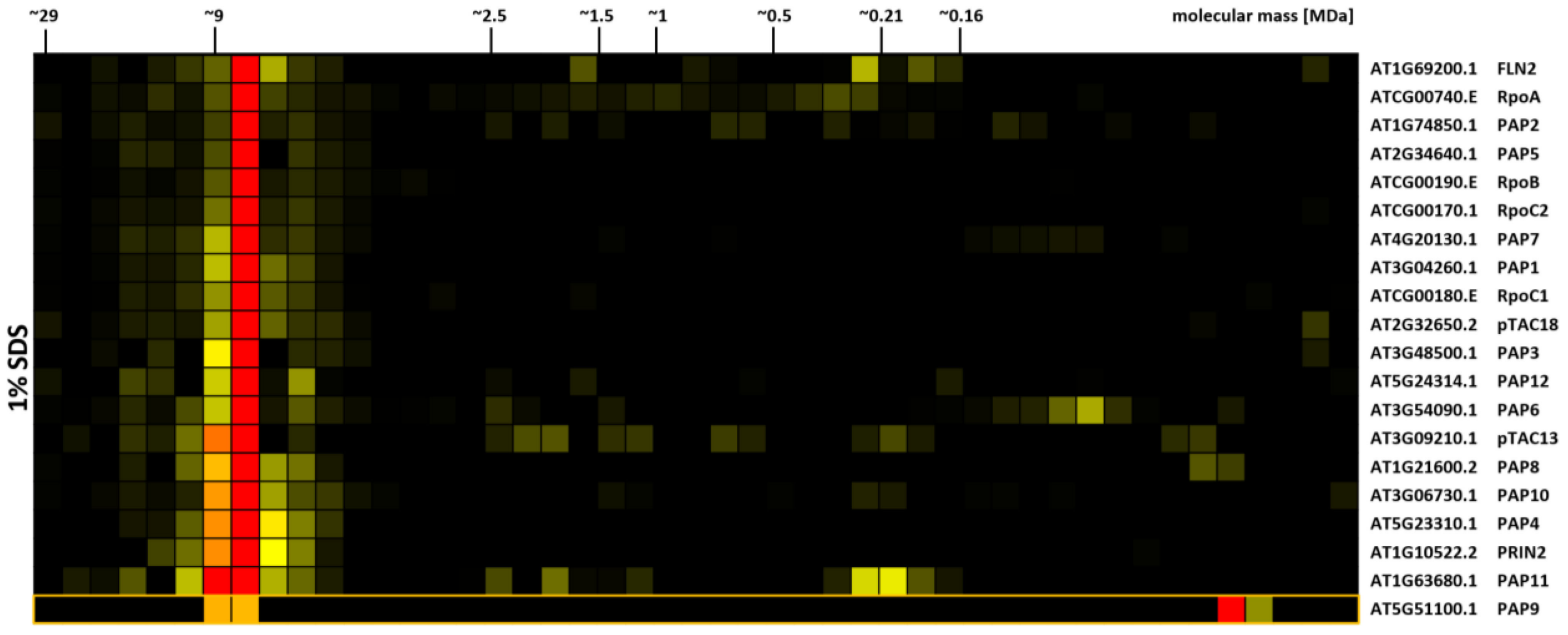
Protein abundance profiles of chloroplast-encoded RNA-polymerase (PEP) subunits. Isolated chloroplasts were treated with 1 mM DSBU, solubilized with SDS, and subjected to the CP workflow as shown in **Supplementary Figure 5**. Protein abundance profiles show high normalized protein abundance in red, low normalized protein abundance in yellow, and non-detectable protein abundance in black. Molecular masses (in MDa) of lpBN-PAGE fractions are indicated at the top. Arabidopsis gene identifiers and protein descriptions are given to the right. The abundance profile of PAP9 was manually added to the cluster and is marked by an orange box.

Within the DSBU cross-linked, SDS-solubilized CP dataset (**Supplementary Figure 8**), one cluster stands out due to its sharpness at a molecular mass of 8–9 MDa (**Figure 5**). The cluster contains all core subunits of the chloroplast encoded RNA polymerase (PEP). In addition, nearly all of its known associated proteins (PAP1–8, 10–12; (Pfannschmidt et al., 2015) are also part of the cluster, as are the recently identified additional PEP subunits FLN2, pTAC18, pTCA13 (Ruedas et al., 2022; Xiong et al., 2022; **Figure 5**). PRIN2, another cluster member, is discussed as a potential component of the PEP complex, since T-DNA insertion mutants lacking PRIN2 show the typical albino to pale green PEP-phenotype. However, by our best knowledge, biochemical data supporting a physical interaction of PRIN2 with the complex have not been presented to this date (Kindgren et al., 2012). The CP-results shown here therefore add further evidence for the presence of this protein within the PEP complex. PAP9 also has a peak at the position of the complex, but additionally shows a strong signal in the low molecular mass region. It thereforte did not cluster with the other subunits. Previously, the PEP complex was reported to possess a much lower mass in the range of 1.0 MDa to 1.1 MDa (Steiner et al., 2011; do Prado et al., 2024; Vergera-Cruces et al., 2024; Wu et al., 2024). However, despite the presence of additional proteins in the cluster, the question why PEP is migrating at a molecular mass distinctively above the previously reported value remains open. CP-results obtained from cross-linked organelles solubilized with DDM and Triton X-100 reveal a complex migrating at ~1 MDa, thus matching previously reported values (**Supplementary Figure 9**). The main factor responsible for the high molecular mass therefore is the cross-linking treatment combined with SDS solubilization (**Supplementary Figure 9**; **Supplementary Table 2**). PEP is an intrinsic part of the plastid transcriptionally active chromosome (pTAC), which is anchored to chloroplast membranes (Pfannschmidt et al., 2015). The high density of nucleic acids, stabilized by their attachment to proteins and membranes, may hinder the complete solubilization of this large protein complex if non-ionic detergents are applied, thus leading to the formation of the ~1 MDa fragment that is resolved after solubilization with DDM and Triton X-100. An association of pTAC13 with both, PEP and chloroplast ribosomes, was recently reported (Xiong et al., 2022), which may serve as a link between the two complexes, effectively bridging transcription and translation. However, abundance of ribosomal proteins in the corresponding fractions is rather low.

### Impact of light intensity on chloroplast PPI patterns

Dynamic chloroplast PPIs are an important component in adjusting the photosynthetic machinery to changing light intensities. Especially PSII complexes and their association with LHCII antenna experience strong changes during acclimation to different light intensities, thereby also influencing the entire thylakoid membrane architecture (Herbstová et al., 2012; Kouřil et al., 2013; Grinzato et al., 2020). PSII and PSI can interact in grana margins, and there is growing evidence that both photosystems associate to form ‘photosystomes’ (Järvi et al., 2011; Suorsa et al., 2015; Rantala et al., 2017; Yokono et al., 2019), which increase in abundance under high light (HL) intensities (Yokono et al., 2015). Besides PSII, PSI also interacts with the NDH complex and has a stabilizing effect on it under enhanced light (EL) conditions (Peng and Shikanai, 2011; Yamori et al., 2015).

**Figure 6:**
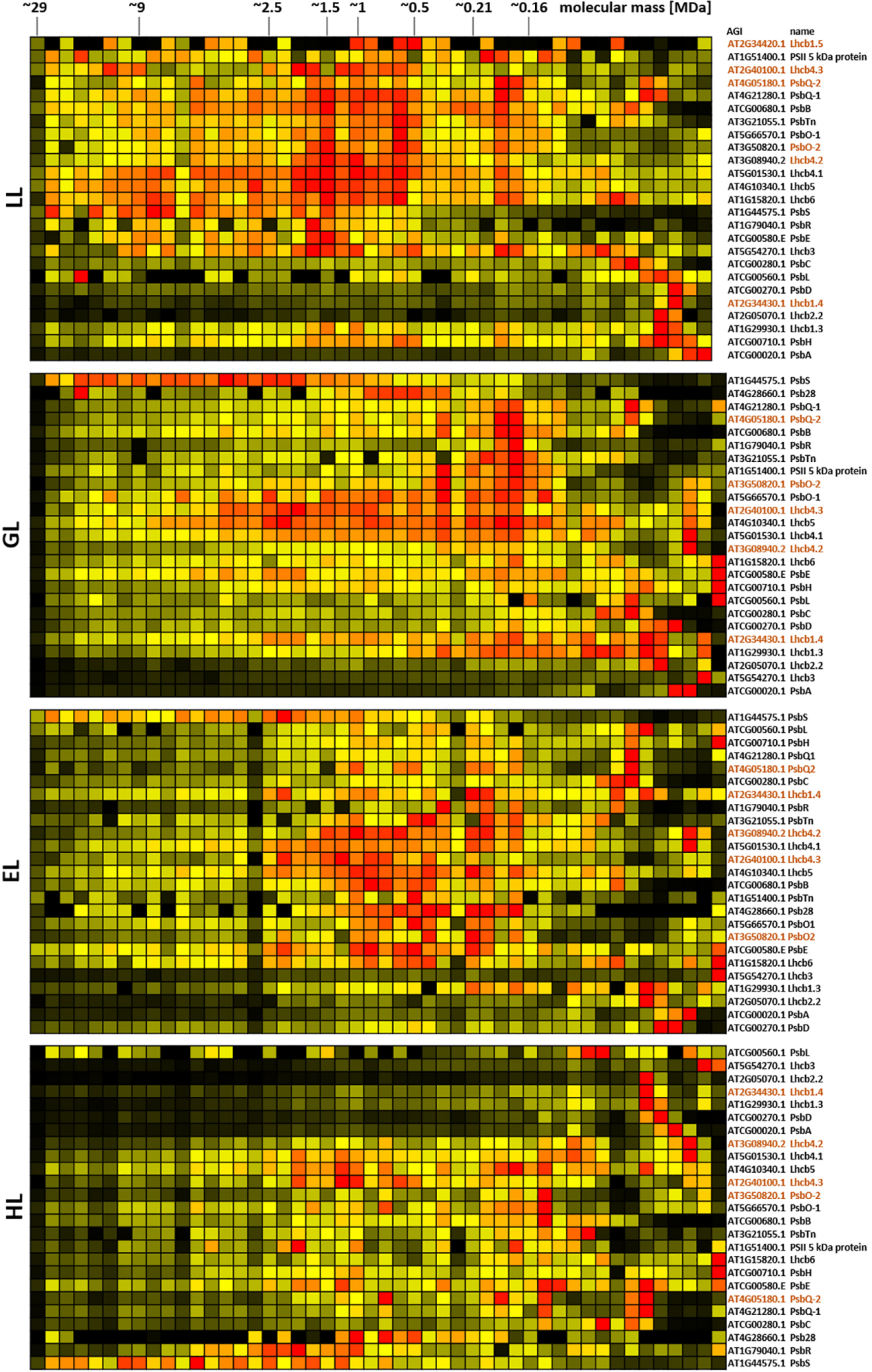
Protein abundance profiles of PSII subunits of leaves after acclimation to different light intensities. Plants grown under increasing light intensities (low light, “LL”, 20 µmol · m^-2^ · s^-1^ ; grow light, “GL”, 120 µmol · m^-2^ · s^-1^; enhanced light, “EL”, 600 µmol · m^-2^ · s^-1^ ; high light, “HL”, 1000 µmol · m^-2^ · s^-1^) were harvested in parallel. PPIs were stabilized before and after chloroplast isolation by cross-linking with 1 mM DSBU. Protein assemblies were subsequently solubilized with 1% [w/v] SDS and subjected to the CP workflow shown in **Supplementary Figure 11**. Abundance profiles of PSII subunits were manually selected and subsequently hierarchically clustered by NOVA. The light treatment is indicated to the left, Arabidopsis gene identifier and protein names are given to the right (isoforms are written in brown). Molecular masses of complexome fractions are given on top in MDa. High protein abundance is indicated in red, medium protein abundance in orange, low protein abundance is depicted in yellow, whereas non detectable protein abundance is displayed in black.

**Figure 7:**
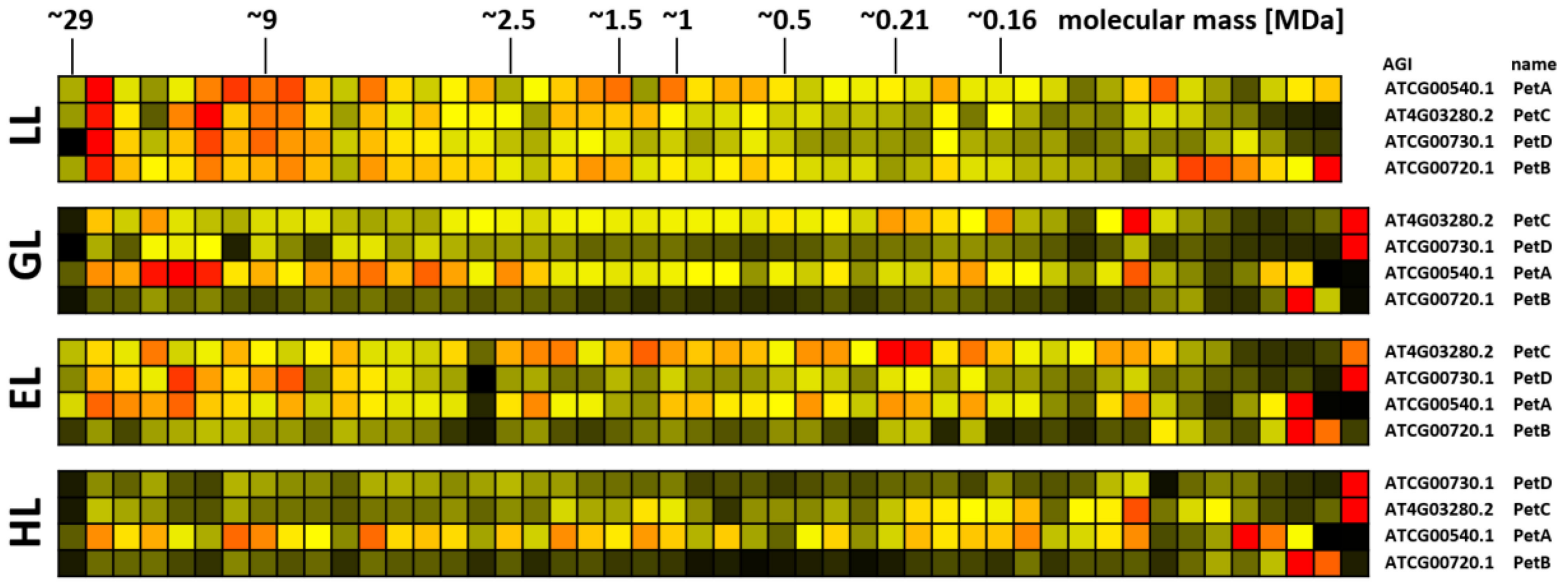
Protein abundance profiles of *b*_6_ *f* subunits after acclimation to different light intensities. Plants grown under increasing light intensities (low light, “LL”, 20 µmol · m^-2^ · s^-1^ ; grow light, “GL”, 120 µmol · m^-2^ · s^-1^; enhanced light, “EL”, 600 µmol · m^-2^ · s^-1^; high light, “HL”, 1000 µmol · m^-2^ · s^-1^) were harvested in parallel. PPIs were stabilized before and after chloroplast isolation by cross-linking with 1 mM DSBU. Protein assemblies were subsequently solubilized with 1% [w/v] SDS and subjected to the CP workflow shown in **Supplementary Figure 11**. Protein assemblies of all treatments were separated on the same lpBN-gel to reduce technical variation. Abundance profiles of complex *b*_6_ *f* subunits were manually selected and subsequently hierarchically clustered by NOVA. The light treatment is indicated to the left, Arabidopsis gene identifier and protein names are given to the right (isoforms written in brown). Molecular masses of complexome fractions are given on top in MDa. High normalized protein abundance is indicated in red, medium protein abundance in orange, low protein abundance is depicted in yellow, whereas non-detectable protein abundance is displayed in black.

**Figure 8:**
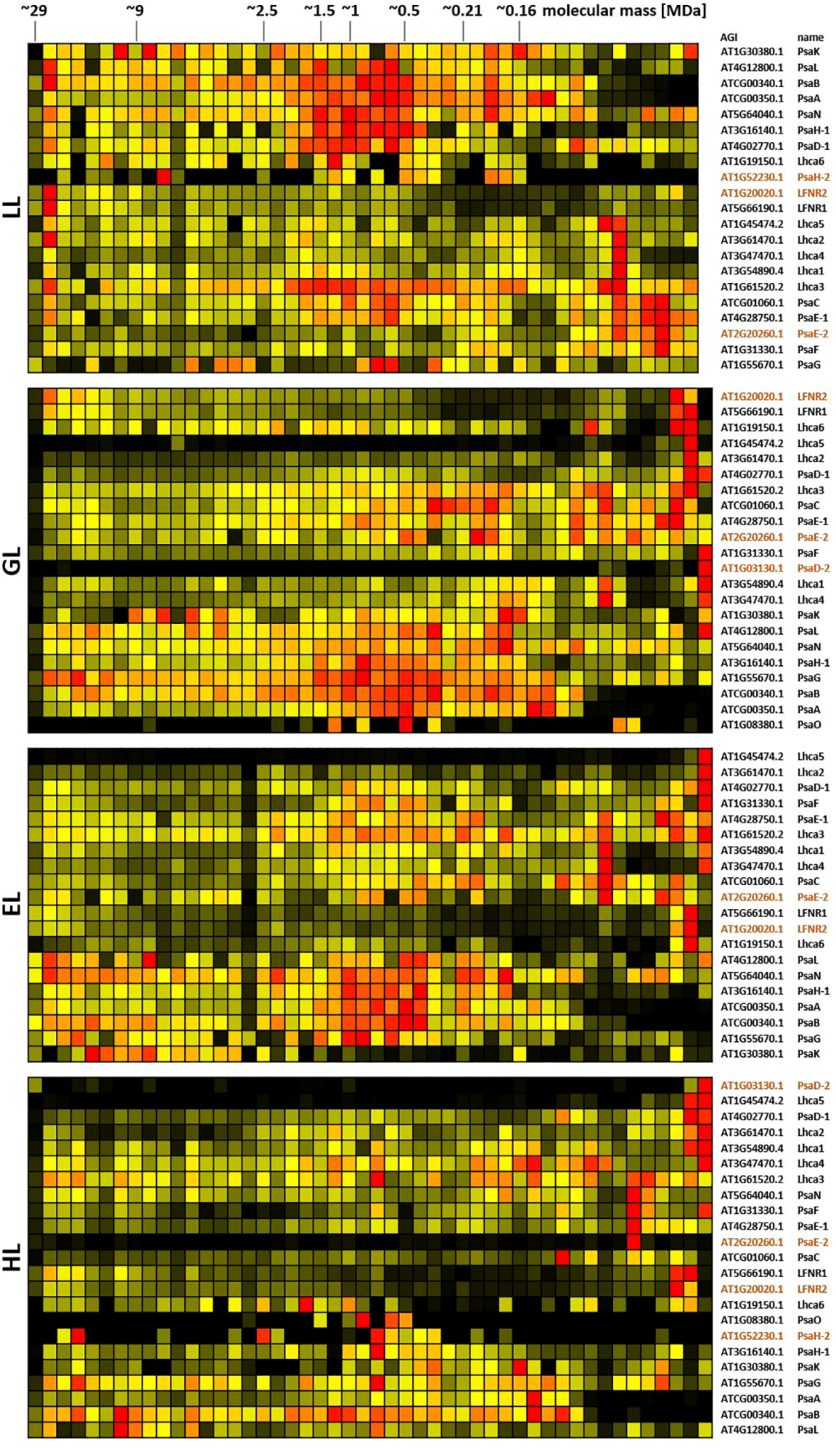
Protein abundance profiles of PSI subunits of leaves acclimated to different light intensities. Plants grown under increasing light intensities (low light, “LL”, 20 µmol · m^-2^ · s^-1^ ; grow light, “GL”, 120 µmol · m^-2^ · s^-1^; enhanced light, “EL”, 600 µmol · m^-2^ · s^-1^; high light, “HL”, 1000 µmol · m^-2^ · s^-1^) were harvested in parallel. PPIs were stabilized before and after chloroplast isolation by DSBU cross-linking. Protein assemblies were subsequently solubilized with 1% SDS and subjected to the CP workflow shown in **Supplementary Figure 11**. Protein assemblies of all treatments were separated on the same lpBN-gel to reduce technical variation. Abundance profiles of PSI subunits were manually selected and subsequently hierarchically clustered by NOVA. The light treatment is indicated at the left, Arabidopsis gene identifier and protein names are given to the right (isoforms written in brown). Molecular masses of complexome fractions are given at the top in MDa. High normalized protein abundance is indicated in red, medium protein abundance in orange, low protein abundance is depicted in yellow, whereas no detected protein abundance is displayed in black.

For deeper insights into the role of chloroplast PPIs in plant acclimation to different light conditions, DSBU-assisted CP using SDS was applied to chloroplasts isolated from plants grown under increasing light intensities (low light, “LL”, 20 µmol · m^-2^ · s^-1^; grow light, “GL”, 120 µmol · m^-2^ · s^-1^; enhanced light, “EL”, 600 µmol · m^-2^ · s^-1^; high light, “HL”, 1000 µmol · m^-2^ · s^-1^; for further details, please refer to the materials and methods section and **Supplementary Figure 10**). To further reduce the loss of PPIs during the organelle isolation procedure, an additional cross-linking treatment was implemented into the workflow directly after cell disruption (**Supplementary Figure 11**). This also necessitated a scaled down organelle isolation procedure to reduce the disruption buffer volume and therefore the total amount of required cross-linker. Together, this led to a more even distribution of signal along the lpBN gel lane, albeit at the cost of a lower overall intensities (**Supplementary Figure 12**). Protein abundance profiles for chloroplast protein complex subunits, either extracted directly from clustered abundance profiles or assembled manually, clearly reacted to altered light intensities (**Figures 6**–**8**; **Supplementary Figures 13**–**15**). Due to the broad distribution of these proteins, a detailed, quantitative assessment of the corresponding results is difficult at this stage, but clear tendencies for PSII, *b*_6_ *f*, and PSI are notable. With increasing light intensity, protein abundance for PSII subunits shifts from higher molecular mass regions towards lower masses (**Figure 6**; **Supplementary Figure 13**). This behavior is pronounced for the Lhcb subunits, which interact dynamically with the PSII core to adjust electron flow to the water-splitting complex in response to demand and supply. One notable exception to this is the PbsS subunit. Involved in non-photochemical quenching, it largely maintains its abundance in the high molecular mass range, most likely in order to protect remaining high molecular mass assemblies of PSII from damage.

Protein abundance of the *b_6_f* complex also reacts to illumination (**Figure 7**). Four subunits (PetA, PetB, PetC, and PetD) were detected in this study, whereas the biochemical properties of the small and highly hydrophobic subunits PetG, PetL, PetM, and PetN hindered identification by MS. Under LL conditions, all four detected subunits peak at ~27 MDa. In plants, interactions of the *b*_6_ *f* complex with other major protein complexes of the plastid ETC chain are currently not reported. However, it seems implausible that a complex with molecular mass of ~210 kDa can increase its molecular mass by factor >100 simply by multimerization. It is therefore hypothesized that the *b_6_f* complex interacts with currently unknown protein partners. Given the functional context in which the *b_6_f* complex operates, other chloroplast electron transfer components are the most probable candidates. With increasing light intensities, protein abundance in this region becomes more and more reduced and protein abundance accumulates more in the low molecular mass range. However, compared to PSII, this effect is less pronounced for the *b_6_f* complex.

PSI subunits, instead, are of highest abundance in the medium mass range under LL condition, but spread out more evenly across the lpBN mass range in higher light intensities (**Figure 8**; **Supplementary Figure 13**). PSI and the NDH complex are reported to form super-complexes consisting of a single NDH complex, to which multiple PSI structures become attached (Otani et al., 2018). This would yield molecular masses of ~1.8 MDa, ~2.4 MDa, ~2.9 MDa, and ~3.5 MDa. Inspection of the PSI and NDH heatmap shows abundance at these molecular masses, but no clear peaks. However, PSI abundance seems to decrease in this mass range at higher light intensities.

In contrast, subunits of the NDH complex and the ATP synthase response less to changing light intensities (**Supplementary Figures 14**, **15**). Notably, both complexes also peak at 27 MDa under LL conditions, similar to PSII, the *b_6_f* complex and PSI. Apart from this, however, no common behavior is recognizable. It is noteworthy that hardly any abundance for the singular 700 kDa NDH complex (Peng and Shikanai, 2011) is detectable in the heatmaps. Instead, protein abundance accumulates from >1 MDa upwards.

In summary, the heatmaps produced by CP from cross-linker treated, isolated Arabidopsis chloroplasts show light dependent changes for ETC complexes PSII, *b*_6_ *f*, as well as PSI, while differences for the ATP-synthase complex and NDH complex subunits are less pronounced. Interestingly, the *b*_6_ *f* complex, PSI, the NDH complex, and the ATP synthase complex all share a common peak at ~27 MDa in LL, suggesting a potential participation within the same mega-structure.

Under low light conditions, the ATP-synthase complex peaks at ~27 MDa, similar to the *b*_6_ *f* complex. In general, the two complexes react similar to increasing light conditions, although the spreading of protein abundance across the fractions only reaches into the low molecular mass region for subunits of the *b*_6_ *f* complex.

**Figure 9:**
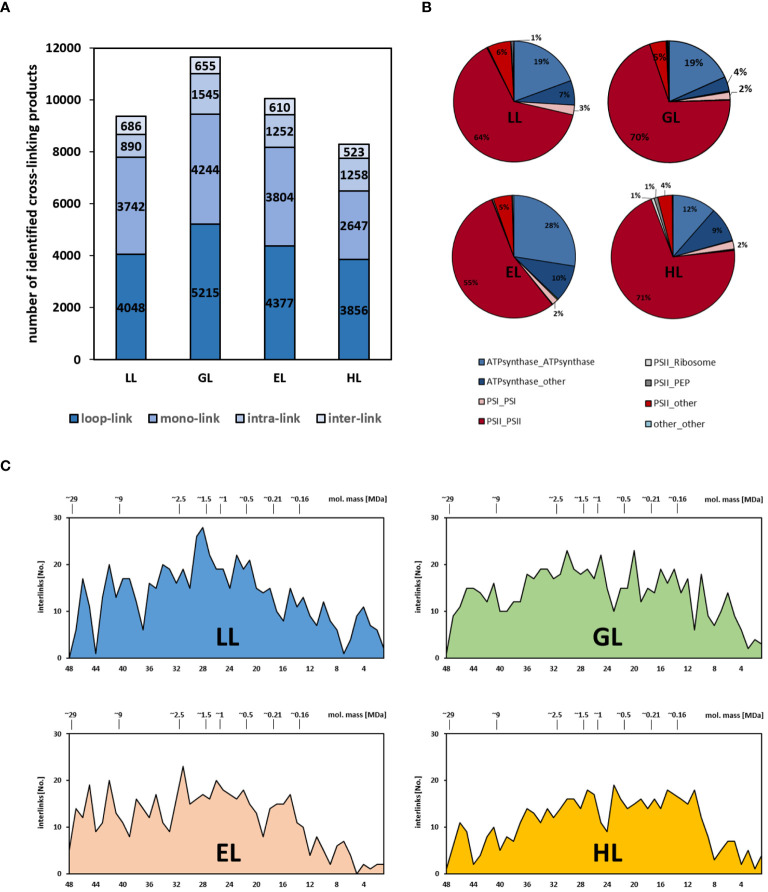
Occurrence and distribution of cross-linking products in response to altered light intensities. **(A)** Cross-linking types across light treatments; **(B)** occurrence of interlinks within chloroplast protein complexes across light treatments; **(C)** interlinks across gel fractions for each light treatment. Numbers on top of graphs indicate electrophoretic mobility (given in MDa), whereas numbers at the bottom denote CP fraction number. Numbers to the left indicate interlink counts for each fraction.

Besides co-migration of proteins in lpBN-PAGE, the cross-linking products identified in the course of the study provide another layer of information on chloroplast PPIs. In total, 2474 interlinks (links between different proteins) are found within the four CP sets (**Figure 9A**; **Supplementary Table 3**), representing 236 unique inter-link species and involving 64 protein groups. These are complemented by 4945 intralinks (cross-linked peptides within the same protein species; 321 of these being non-redundant), 14437 looplinks (cross-linked amino acids within the same peptide species) and 17496 monolinks (only one end of the cross-linker reacting with a single peptide). However, since this study focusses on PPIs, only interlinks are considered here. Unique interlinks are detected in each light treatment (**Table 1**; **Supplementary Table 3**). The same interlinks are often found in two or more light treatments, albeit in differing numbers (**Table 1**; **Supplementary Figure 16**). Between the individual datasets, total numbers of identified interlinks are comparable, but show a tendency towards lower identification rates at higher light intensities (**Figure 9A**; **Supplementary Table 3**). The bulk of interlinks is found between PSII subunits, followed by the ATP-synthase complex, which also contains a substantial amount of the identified interlinks. Only small numbers of interlinks were identified between PSI subunits. For PSII and the ATP-synthase complex, interlinks between the subunits of each of these complexes and other proteins were also found (**Figure 9B**). Distribution of cross-links over the mass range of the gel is fairly even (**Figure 9C**), but a reduction of hits in the high molecular mass region is observable with increasing light intensities. Since the majority of interlinks is PSII-related, these results largely match the protein abundance shift for PSII subunits (**Figure 6**).

**Table 1:** Non-redundant interlinks of PSII and FtsH subunits with external proteins.

AGI prot. 1	prot. 1	AGI prot. 2	prot. 2	LL	GL	EL	HL	sum
**ATCG00270.1**	**PsbD**	**AT5G02160.1**	**FIP5**	**5**	**14**	**10**	**0**	**29**
AT2G20890.1	Psb29	AT5G42270.1	FtsH5	0	3	11	0	14
ATCG00680.1	PsbB	AT3G26710.1	CCB1	1	1	2	1	5
ATCG00680.1	PsbB	AT5G65020.2	ANNAT2	5	0	0	0	5
ATCG00270.1	PsbD	ATCG00170.1	RpoC2	0	0	2	2	4
AT1G06680.1	PsbP1	AT2G27530.2	RPL10AB	0	2	0	0	2
AT4G10340.1	Lhcb5	AT5G57490.1	VDAC4	2	0	0	0	2
AT4G24280.1	HSP70–6	ATCG00580.E	PsbE	1	1	0	0	2
**AT5G02160.1**	**FIP5**	**ATCG00580.E**	**PsbE**	**2**	**0**	**0**	**0**	**2**
ATCG00580.E	PsbE	AT2G24820.1	Tic55	0	2	0	0	2
ATCG00680.1	PsbB	AT4G37820.1	UFP	0	2	0	0	2
**ATCG00680.1**	**PsbB**	**AT5G02160.1**	**FIP5**	**1**	**0**	**1**	**0**	**2**
AT1G03600.1	PSB27-H1	AT1G12250.2	TL20.3	0	1	0	0	1
AT1G03600.1	PSB27-H1	AT1G36240.1	RPL30A	0	1	0	0	1
AT1G50250.1	FtsH1	AT3G55280.3	RPL23AB	0	0	1	0	1
AT1G79040.1	PsbR	AT4G04020.1	FIB1A	0	0	0	1	1
AT2G30950.1	FtsH2	AT2G20890.1	Psb29	0	1	0	0	1
AT3G25920.1	RPL15	AT3G50820.1	PsbO2	0	0	0	1	1
AT3G50820.1	PsbO2	ATCG01100.1	NdhA	0	0	1	0	1
AT4G24280.1	HSP70–6	ATCG00730.1	PetD	0	1	0	0	1
AT5G07090.2	RPS4B	ATCG00710.1	PsbH	0	0	1	0	1
ATCG00270.1	PsbD	AT1G74970.1	RPS9	1	0	0	0	1
ATCG00270.1	PsbD	AT4G38780.1	PRP8B	1	0	0	0	1
ATCG00560.1	PsbL	AT5G07090.2	RPS4B	0	0	1	0	1
ATCG00680.1	PsbB	AT1G18170.1	FKBP17–2	0	0	0	1	1
ATCG00680.1	PsbB	AT5G67500.1	VDAC2	0	0	0	1	1
ATCG00710.1	PsbH	AT4G11010.1	NDPK3	0	1	0	0	1

AGI prot. 1, Arabidopsis gene identifier of the first interaction partner; prot. 1, name of the first interaction partner; AGI prot. 2, Arabidopsis gene identifier of the second interaction partner; prot. 2, name of the second interaction partner; LL, quantity of identifications across all fractions of the LL complexome; GL, quantity of identifications across all fractions of the GL complexome; EL, quantity of identifications across all fractions of the EL complexome; HL, quantity of identifications across all fractions of the HL complexome; sum, sum of identifications across all sets; UFP, unknown function protein. FIP5-interactions with PSII subunits are highlighted in bold writing.

Interestingly, no interlinks were detected for several other chloroplast protein complexes, i.e. the RubisCO-complex, the *b*_6_ *f* complex, or the NDH-complex. We currently have no explanation for this, but (with the obvious exception of RubisCO) speculate that this effect is related to protein abundance. Lower abundance of a given protein assembly will result in lower amounts of detectable cross-linking products. Given that the percentage of cross-links within the possible cross-linking space is quite low (usually ~1–5%; Steigenberger et al., 2020), interlinks within lower abundant protein assemblies will be under-represented (**Table 1** and **Supplementary Table 3**) due to the sensitivity limits of the MS detector. This however, does not mean that the stability of these assemblies is not increased by cross-linking treatment. Even if not detectable, cross-links are nevertheless expected to be present in these protein assemblies and should confer stability in the presence of detergents.

### New insights into the composition of the PSII super-complex and its repair machinery

Although the majority of identified interlinks in each CP set are between individual PSII subunits, some are also between PSII subunits and proteins not directly related to primary PSII functions. These interactions are potentially interesting, as they foster identification of unknown players in PSII function and maintenance. For deeper insights into PSII physiology, all interlinks were filtered for cross-links in which only one partner is a PSII subunit (or a protein participating in PSII assembly and degradation; **Table 1**). Interestingly, most interlinks involving PSII are between the thylakoid-localized *FtsH5 interacting protein* (FIP5) and the PSII core subunits PsbB, PsbD and PsbE. FIP5 and FtsH5 are both involved in the exchange of the D1 (PsbA) subunit, which is susceptible to photo-oxidative damage.

**Figure 10:**
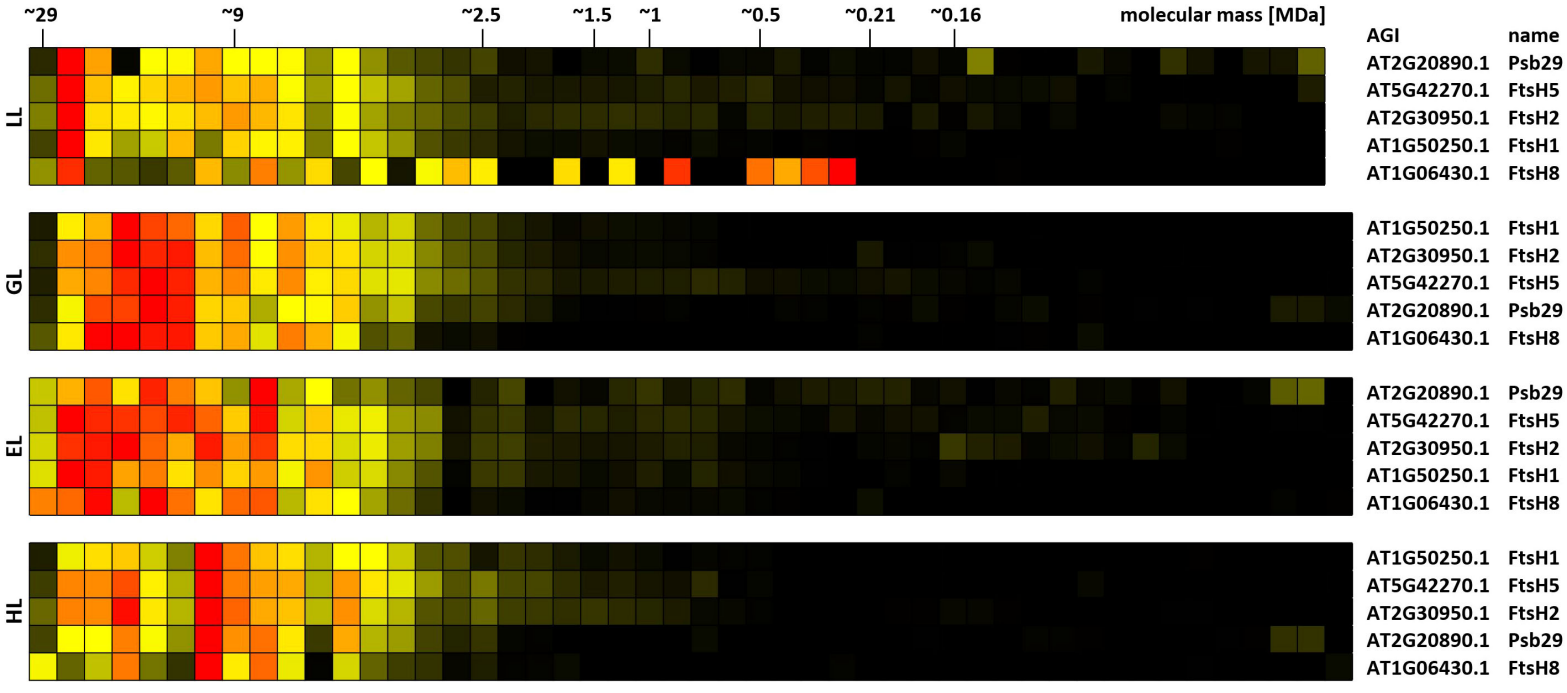
Clustered protein abundance profiles of Psb29 and FtsH complex subunits of leaves at different light intensities. Plants grown under increasing light intensities (low light, “LL”, 20 µmol · m^-2^ · s^-1^ ; grow light, “GL”, 120 µmol · m^-2^ · s^-1^; enhanced light, “EL”, 600 µmol · m^-2^ · s^-1^; high light, “HL”, 1000 µmol · m^-2^ · s^-1^) were harvested in parallel. PPIs were stabilized before and after chloroplast isolation by DSBU cross-linking. Protein assemblies were subsequently solubilized with 1% SDS and subjected to the CP workflow shown in **Supplementary Figure 11**. Protein assemblies of all treatments were separated on the same lpBN-gel to reduce technical variations. Abundance profiles of Psb29 and FtsH proteins were manually selected from the heatmaps shown in **Supplementary Figures 17**–**20** and subsequently clustered hierarchically. The light treatment is indicated to the left, Arabidopsis gene identifier and protein names are given to the right. Molecular masses of complexome fractions are given on top in MDa. High normalized protein abundance is indicated in red, medium protein abundance in orange, low protein abundance is depicted in yellow, whereas non-detectable protein abundance is displayed in black.

FIP5 was originally identified in a screen for proteins involved in abiotic stress responses. T-DNA insertion mutants lacking FIP5 are characterized by increased resistance against HL (Lopes et al., 2018), suggesting that PSII-associated FIP5 blocks access of FtsH to damaged D1 protein. Indeed, the number of identified cross-links between FIP5 and PSII declines with increasing light intensities (**Table 1**; **Supplementary Table 3**). In a large-scale yeast-two-hybrid experiment FIP5 was found to interact with FtsH5 as bait (Lopes et al., 2018). Interlinks between FIP5 and FtsH5 were not detected during the course of this study. Instead, interlinks between FtsH2/Ftsh5 and Psb29 are found (**Table 1**). Psb29 is suggested to play a role in PSII biogenesis and also promotes association of LHCII complexes with PSII (Keren et al., 2005; Huang et al., 2013). 2D-BN/SDS-PAGE of cyanobacteria revealed co-migration of Psb29 and FTSH proteins and Psb29 is also expected to interact with FtsH in plants (Bec̆ková et al., 2017). However, to our knowledge, no biochemical evidence for this is currently available. *A. thaliana* mutants lacking Psb29 are characterized by a variegated leaf phenotype (Keren et al., 2005), closely resembling the phenotypes of plants lacking either FtsH2 or FtsH5 (Sakamoto et al., 2002). Psb29 therefore not only associates with the thylakoid FtsH complex, but rather seems to be an essential component conveying functionality to the complex. A permanent association of Psb29 to the FtsH complex is also supported by similar migration profiles of Psb29 and subunits of the FtsH complex, as well as their respective response to altered light intensities (**Figure 10**). Under LL, all FtsH subunits peak in a high molecular mass cluster of ~22 MDa, whereas protein abundance shifts towards lower fractions under increasing light intensities. It is therefore suggested that plant Psb29 is an FtsH-associated protein involved in PSII maintenance.

### A web-based tool for analyzing XL-CP datasets

**Figure 11:**
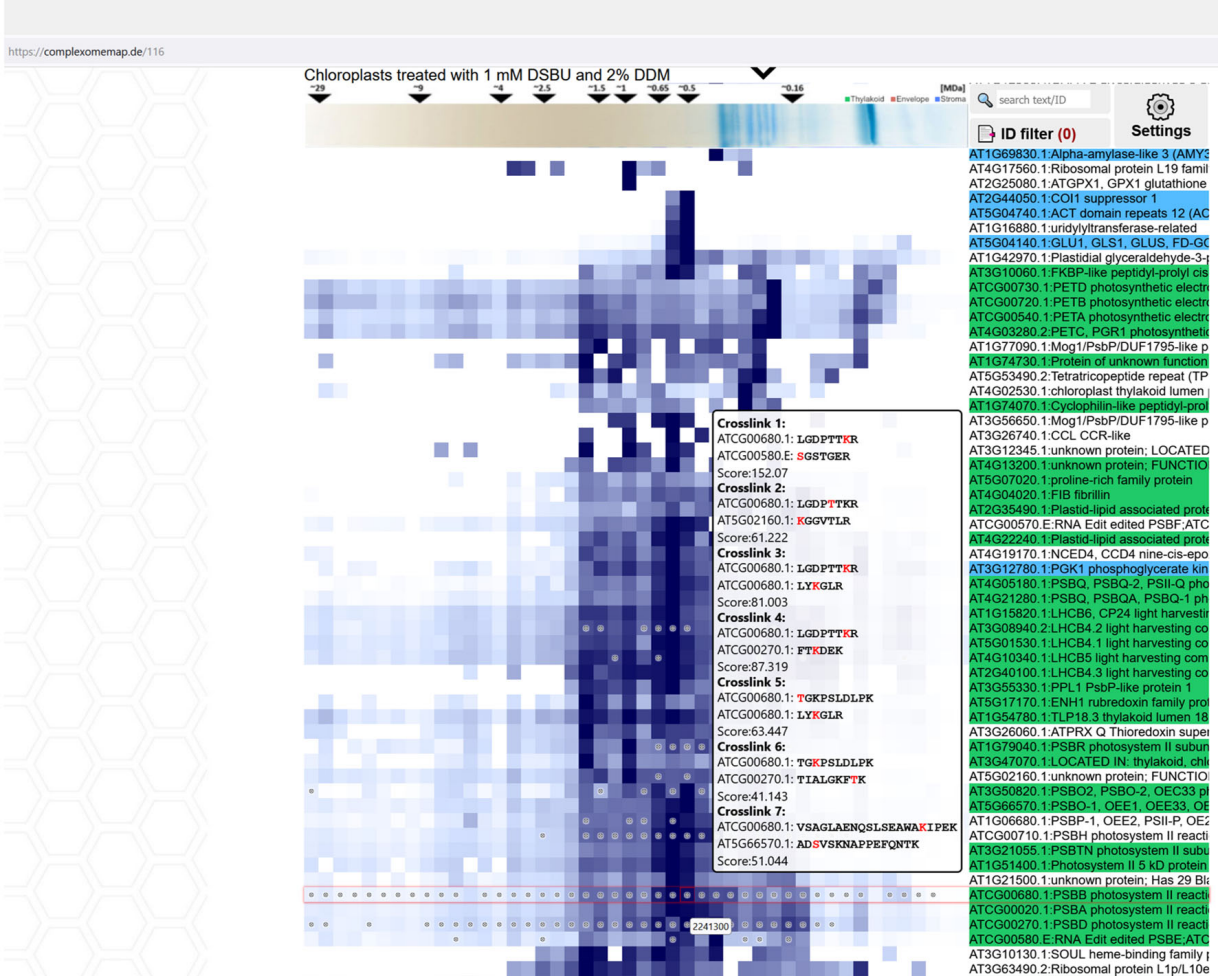
Interactive Complexome Map featuring a build-in cross-link viewer. Each fraction containing detected cross-links is marked with a circle. The cross-link viewer enables intuitive exploration of detected cross-links in each complexome set by hovering the cursor over the selected field in abundance profiles. A prompt then displays the cross-linked peptide sequences with their respective AGIs, cross-link score, and cross-linked amino acids highlighted in red. CP-Maps are accessible at https://complexomemap.de/projects-interaction-chloroplasts.

During the course of the study, 17 complexome maps have been produced, each of them comprising several hundreds to more than thousand proteins and, in most cases, also cross-linking MS data. A full presentation of these data within the limited space available here is not possible. As such, we here focus on aspects, which we deemed to be of potential interest for a broader audience, thereby inevitably ignoring large parts of the data. To enable free access to the hidden information in our data, a searchable web-resource integrating XL-MS data and protein abundance profiles has been created (**Figure 11**). The browser interface allows manual inspection of each of the complexome maps, provides a search tool for proteins of interest, and enables comparison of abundance profiles for selected proteins. Information on the presence and identity of cross-linking products is provided as well. By clicking on identified interlinks, a new window displaying all interaction partners identified by cross-linking will open. Where applicable, color-coding assigns proteins to their chloroplast sub-compartment (envelope, stroma or thylakoids; data obtained from http://at-chloro.prabi.fr:8080/at_chloro/ (Ferro et al., 2010). The tool is accessible at https://complexomemap.de/projects-interaction-chloroplasts/. We invite scholars to use this tool for data mining purposes.

## Outlook

The workflow established here is an advancement of classical CP, using MS-cleavable cross-linkers to stabilize protein complexes during organelle isolation and separation of protein assemblies by lpBN-PAGE. As such, it provides additional, quantifiable MS information on protein proximity via detectable peptide interlinks. However, although the number of identified cross-links, as well as the ratio between inter-and intralinks, is within the range of previous reports (Klykov et al., 2018; Stieger et al., 2019; Albanese et al., 2020; Hevler et al., 2021), it is obvious that this aspect leaves room for further improvement. Fractionation by size exclusion or strong cation exchange chromatography is frequently used to enrich cross-linked peptides in order to boost their numbers (Iacobucci et al., 2018). Cross-linkers equipped with an affinity tag also are promising to increase yield of cross-linked peptides and their identification (Steigenberger et al., 2019). In combination with CP, such an enrichment step and the extra MS-analysis which would be needed to follow, dictates a considerable increase of MS time, since each fraction would need to be analyzed twice (input and enriched). The gain in data quality achievable by the introduction of cross-linking to classical CP workflows and the ever-increasing speed and sensitivity of modern MS-systems are expected to overcompensate the issue of additionally required MS run-time and may therefore be worth the investment. However, even in the absence of detectable quantities of cross-linking products, cross-linking stabilizes fragile PPIs and assists in maintaining the *in vivo* chloroplast PPI landscape during organelle preparation and electrophoresis. As such, the use of MS-cleavable cross-linkers is advisable for future CP applications. As exemplified by the reaction of electron transfer components to light intensity, the combination of cross-linking and CP is able produce quantitative PPI data. For research on chloroplast PPIs, it therefore lends itself to comparative analyses, for example between WT and loss-of-function mutants, plants subjected different growth conditions, or between chloroplasts in varying developmental stages. CP-based comparisons between chloroplasts isolated from different cell types, which might become achievable by new organelle-tagging strategies (Boussardon et al., 2023), might be another exciting future application.

## Experimental procedures

### Plant material and cultivation

*Arabidopsis thaliana* (ecotype Columbia 0) plants were grown in soil (Einheitserde Special Profisubstrat, Patzer Erden GmbH, Sinntal-Altengronau, Germany) under a photosynthetically active photon flux density (PPFD) of 120 µmol · m^-2^ · s^-1^ (using Philips F25T8/TL841 fluorescence lamps). Plants were grown for five weeks under long day conditions (16 h light/8 h darkness) at 22°C in the light and 20°C in the dark. For different light treatments, plants were illuminated for 8 h under 300 W LED lamps at PPFDs of 20, 120, 600 and 1000 µmol · m^-2^ · s^-1^, in the following referred to as low light (LL), growth light (GL), enhanced light (EL) and high light (HL), respectively. Spectral distributions of the light applied during these treatments are shown in **Supplementary Figure 10**. Chloroplasts acclimated to different PPFDs were isolated in parallel and separated in the same lpBN-PAGE to avoid technical variation.

### Mitochondria isolation and solubilization

Freshly isolated mitochondria from *A. thaliana* leaves were obtained as reported previously (Rugen et al., 2021). Protein concentration was adjusted to 3 µg ∙ µL^-1^ before solubilization with 2.5% [w/v] digitonin. Approximately 125 µg mitochondrial protein (according to Bradford) was used for lpBN-PAGE.

### Chloroplast purification

All materials and buffers were cooled to 4°C before use and each step was performed at 4°C or on ice. 15–30 g of healthy looking rosette leafs were harvested 8 h after the onset of illumination and supplemented with 13.3 ml ∙ g^-1^ homogenization buffer (2 mM EDTA; 1 mM MgCl_2_; 5 mM sodium ascorbate; 10 mM NaF; 330 mM sorbitol; 0.5% [w/v] BSA; 50 mM HEPES-KOH, pH 8.0). Cell disruption was performed in a blender (Warring pro, Warring, Stamford, U.S.A.) for 3 s at high speed, followed twice by 3 s at low speed. In between each of these steps, plant material was allowed to sediment for 30 s. The homogenate was filtered through two layers of Miracloth (Merck KGaA, Darmstadt, Germany) and centrifuged for 5 min at 300xg. The pellet was resuspended in 2 mL homogenization buffer and loaded onto a 10 mL self-forming Percoll gradient (35% [v/v] Percoll; 10 mM NaF; 330 mM sorbitol; 50 mM HEPES-KOH, pH 8.0). After 20 min centrifugation in a swing-out rotor (Surespin 630 x 17; Thermo Fischer Scientific, Dreieich, Germany) at 67117xg, intact chloroplasts were carefully recovered with a Pasteur pipette from the lower green band of the gradient. Chloroplasts were washed once in 25 ml wash buffer (330 mM sorbitol; 10 mM NaF; 50 mM HEPES-KOH, pH 8.0) at 1000xg for 5 min. Chloroplast pellets were subsequently resuspended in 100–500 µL of wash-buffer. Chlorophyll concentration was determined according to Arnon (1949) and subsequently adjusted to 0.3 µg ∙ µL^-1^. To optimize cross-linking of chloroplast proteins, DSBU was dissolved in anhydrous DMSO at different concentrations (5 mM to 50 mM). The resulting cross-linking solution was supplemented to freshly isolated chloroplasts yielding a final DMSO concentration of 10% [v/v] and was allowed to incubate for 2 h at room temperature.

### Chloroplast mini-preparation

To implement an early DSBU cross-linking step directly after cell disruption, 2 g of leaf material were lysed in 2 ml homogenization buffer. Lysis was performed for 5 min using mortar and pestle, followed by filtration through a 10 µm nylon mesh. The filtrate was supplemented with cross-linking solution containing 10 mM DSBU in anhydrous DMSO (yielding a final concentration of 10% [v/v]) and was allowed to incubate for 2 h. The following steps were performed as described above.

### Solubilization of chloroplast proteins

Samples destined for solubilization with digitonin, Triton X-100, and SDS were 1:1 diluted in 100 µL of lpBN-solubilization buffer (50 mM NaCl_2_; 2 mM aminocaproic acid; 1 mM EDTA; 50 mM imidazole-KOH, pH 7.0) containing different detergent concentrations. For digitonin, final concentrations between 2.5–5% [w/v] were selected, Triton X-100 was supplemented using final concentrations between 1%-16% [v/v], and for SDS final concentrations between 0.5 - 8% [w/v] were chosen. After incubation for 20 min at room temperature, samples were centrifuged for 2 mins at 20000xg. Except for SDS-treated samples, supernatants were supplemented 5% [v/v] Serva Blue G solution (750 mM aminocaproic acid; 5% [w/v] Coomassie brilliant blue G250) to introduce negative charges for subsequent electrophoretic separation. For DDM solubilization, 80 µL sample were mixed with 20 µL 10% [w/v] DDM, yielding a final concentration of 2% [w/v].

### Large pore (Blue) Native-PAGE

LpBN-PAGE was performed according to (Rugen et al., 2021) with minor modifications to adapt electrophoretic separation to chloroplast protein complexes. Chloroplast proteins equivalent to 20 µg of chlorophyll were loaded on each lane. A voltage gradient from 100–500 V was applied for 10 h, followed by another 10 h at 500 V. In both steps, the current was limited to 15 mA. For separation of protein complexes solubilized with SDS, the cathode buffer contained no Coomassie, but 0.1% [w/v] SDS instead. During the first 45 mins of the run 10a gradient from 0 to 100 V was applied, followed by a second, 18 h gradient from 100 to 460 V. Gel staining was performed using the Coomassie colloidal staining procedure (Neuhoff et al., 1988). Fractionation of gel lanes and subsequent tryptic in-gel digestion was performed as outlined in (Rugen et al., 2021).

### Liquid chromatography coupled tandem mass spectrometry (LC-MS/MS)

Initial experiments were performed using an Ultimate 3000 UHPLC system coupled to an Orbitrap Q-Exactive mass spectrometer (both Thermo Fischer Scientific, Dreieich, Germany). Tryptic peptides were resuspended in 5% [v/v] acetonitrile (ACN) and 0.1% [v/v] trifluoracetic acid (TFA). Approximately 200 ng of peptides were injected into a 20 µL sample loop, before being loaded at 3 µL · min^-1^ onto a 2 cm C18 reverse phase trap column (Acclaim PepMap 100, Thermo Fischer Scientific, Dreieich, Germany) with an inner diameter of 75 µm, particle size of 5 µm and a pore size of 100 Å. Separation of peptides was achieved using a flow of 0.25 µl ∙ min^-1^ over a 50 cm C18 reverse phase analytical column (Acclaim PepMap 100, Thermo Fischer Scientific, Dreieich, Germany) with an inner diameter of 75 µm, particle size of 3 µm, and a pore size of 100 Å in a 60 min non-linear 5–36% [v/v] ACN gradient (in 0.1% [v/v] formic acid; FA) at a column temperature of 45°C. Eluting peptides were transferred into the MS by electrospray ionization (ESI) using a NSI source equipped with a stainless steel nano-bore emitter (both Thermo Fischer Scientific, Dreieich, Germany). MS settings were selected as outlined in (Rugen et al., 2021)

Additional to the standard shotgun settings described above, parameters were adapted to improve detection of cross-linked peptides. Voltage was set to 2.2 kV, capillary temperature to 275°C and the RF-level of the S-lens to 50%. The MS was operated in positive ion mode and a data dependent acquisition (DDA) strategy was used to record top ten MS/MS spectra. High-resolution full MS scans were recorded at a resolution of 140000 and a scan range of 300 to 1700 m/z. The automated gain control (AGC) target was set to 3e6 and the maximum injection time to 100 ms. For MS/MS scans, top 10 peptides were fragmented using a stepped fragmentation with normalized collision energies (NCE) of 27%, 30% and 33%. MS/MS scans were acquired at a resolution of 17500 and an isolation window of 2 m/z. The maximum injection time was set to 250 ms at an AGC target of 2e3. Only ions with 3 to 8 positive charges were considered at a dynamic exclusion of 60 s. These specific MS settings are referred to as ‘Orbitrap XL-settings’ in this publication.

Most samples were analyzed by timsTOF-MS as described in Klusch et al. (2022). For this, peptides were resuspended in 0.1% [v/v] formic acid and approximately 200 ng of peptides were injected into a nanoElute HPLC coupled to a timsTOF Pro instrument (both Bruker Daltonics GmbH & Co KG, Bremen, Germany). Samples were stored in a 20 µL sample loop before being loaded on a 5 mm reverse phase C18 PepMap 100 trap-column (Thermo Fischer Scientific, Dreieich, Germany) with a diameter of 0.3 mm, a particle size of 5 µm, and a pore size of 100 Å at a constant pressure of 500 bar. Peptides were separated on a 10 cm reverse phase C18 Bruker TEN analytical column (Bruker Daltonics GmbH & Co. KG, Bremen, Germany) with an inner diameter of 75 µm, particle size of 1.9 µm and a pore size of 120 Å at a flow rate of 500 nl ∙ min^-1^ and a column temperature of 50°C. Elution of peptides was achieved using a 17.8 min non-linear 2% [v/v]-30% [v/v] ACN gradient in 0.1% [v/v] FA. The pre-installed method ‘DDA PASEF 1.1sec_cycletime’ was used for data acquisition.

Additionally, the pre-installed method for analysis of cross-linked peptides ‘Bruker_Xlink_default’ was used. Ionization of peptides was done using a captive spray source (Bruker Daltonics GmbH & Co. KG, Bremen, Germany) at an end plate offset of 500 V, capillary voltage of 4.5 kV and a temperature of 180°C. The nebulizer pressure was set to 0.4 bar while the dry gas flow was kept at 3 L ∙ min^-1^. TIMs was enabled in a parallel accumulation serial fragmentation (PASEF) protocol adapted to the enrichment of cross-linked peptides. Ions with positive charges from 3 to 8 within a m/z range from 100–1700 m/z were considered for TIMs ramping and subsequent fragmentation. Base values for mobility dependent energy ramping were set to 85 eV at an inverse reduced mobility (1/K_0_) of 1.63 V ∙ s^-1^ ∙ cm^-2^ and 25 eV at 0.73 V ∙ s^-1^ ∙ cm^-2^. Collision energies were linearly interpolated between these values. TIMs stepping was activated to merge two TIMs scans recorded with 85% and 115% of the collision energy profile into a single PASEF MS/MS frame. Ramp time was set to 166 ms, transfer time to 60 µs and the pre-pulse storage time to 12 µs, active exclusion was set to 0.4 min. The target intensity was set to 40000 to improve spectral quality. Seven PASEF ramps were applied in a single cycle, resulting in a cycle time of 2.58 s. The TIMs device was calibrated after each experiment using filter-lock masses and the auto-calibration protocol.

A nanoElute 2 HPLC (Bruker Daltonics GmbH & Co KG, Bremen, Germany) was used to re-analyze selected samples. Approximately 200 ng of peptides in 0.1% [v/v] formic acid were stored in a 20 µL sample loop before loading on a 15 cm reverse phase Aurora Elite CSI analytical column (IonOpticks PTY LPD, Fitzroy, Australia) with an inner diameter of 75 µm, particle size of 1.7 µm and a pore size of 120 Å at a flow rate of 500 nl ∙ min^-1^ and a column temperature of 50°C. Elution of peptides was achieved using a 17.8 min non-linear 5% [v/v]-30% [v/v] ACN gradient in 0.1% [v/v] FA. Peptides were ionized at a capillary voltage of 1.6 kV and a temperature of 180°C. Ions with a positive charge from 0 to 5 with a m/z range from 100–1700 m/z were considered for TIMs ramping. The pre-installed method ‘DDA PASEF-short_gradient_0.5sec_cycletime’ was used for data acquisition. Four PASEF ramps with a 1/K_0_ start of 0.85 V ∙ s^-1^ ∙ cm^-2^, a 1/K_0_ end of 1.30 V ∙ s^-1^ ∙ cm^-2^, ramp time of 100 ms and an accumulation time of 100 ms were chosen, resulting in a total cycle time of 0.53 s. Target intensity was set to 20000 with an intensity threshold of 2500 and active exclusion of 0.4 min. Collision energies were set to 20 eV for a 1/K_0_ value of 0.6 V ∙ s^-1^ ∙ cm^-2^ or to 59 eV for a 1/K_0_ value of 1.6 V ∙ s^-1^ ∙ cm^-2^ and were linear interpolated between these values.

An overview summarizing which MS system and settings were used for acquisition of individual complexome maps is included to the raw data at the MassIVE depository with the identifier: MSV000091808.

### Processing of MS data

MS raw files were queried against an in-house modified TAIR10 database using MaxQuant (Cox and Mann, 2008) versions 2.0.1.0 to 2.1.4.0. Datasets that are compared against each other were always analyzed with the same MaxQuant version and settings. Detailed information regarding the MS system, settings and MaxQuant versions used for each experiment can be found at the MassIVE MS data deposit with the identifier MSV000091808. Each fraction was considered as an individual experiment by using the ‘no fractions’ function. Oxidation of methionine and acetylation of the N-terminus were selected as variable modifications, whereas carbamidomethylation of cysteine residues was chosen as a fixed modification. Specific digestion was set to trypsin. If cross-linking was applied, three missed cleavages were allowed, whereas a maximum of two missed cleavages was allowed for untreated samples. The maximum charge was set to eight for cross-linked samples and to four if no cross-linking was applied. Minimum peptide length was set to six for analyses of cross-linked samples and to seven for samples without cross-linking treatment. Maximum peptide mass was set to 6000 Da for cross-linked samples, whereas it was 4600 Da for untreated samples. A false discovery rate (FDR) of 1% was allowed at both the peptide spectrum match (PSM) and protein level. Semi-quantitative protein identification was done using the intensity based abundance quantification (iBAQ) function. Additionally, the ‘match between runs’ function was always enabled, whereas the ‘refine peak’ function was only enabled for raw files obtained with the Q-Exactive instrument.

Identification of cross-linked peptides was done using the MaxLynx feature of MaxQuant versions 2.0.2.0 and 2.1.0.0 (Yılmaz et al., 2022). The search space was limited to proteins that were identified in corresponding samples in a preliminary MaxQuant run. DSBU-2 was selected as a MS2-cleavable cross-linker, with a minimum cross-linked peptide length of 6, the minimum partial score for cross-linked peptides was set to 10. Minimum matches was set to 3 and ‘separate protein intra-and inter-cross links’ was activated. Remaining MaxQuant settings were selected as described above for cross-linked samples, with the only exception that no iBAQ calculation was performed.

Contaminants were manually removed before complexome maps based on iBAQ values were produced using the NOVA software (Giese et al., 2015; version 0.5.8). Hierarchical clustering of abundance profiles was performed using the ‘average linkage’ and ‘Pearson correlation distance’ function.

### Determination of photosystem stoichiometry

The PSII-PSI ratio was calculated according to McKenzie et al., 2020, with minor adjustments. First, abundance of photosynthetic complexes was computed by cumulating iBAQ values of each subunit across all lpBN-PAGE fractions. These cumulated iBAQ values were then summed up for all identified subunits of a complex. The result was subsequently divided by the number of identified subunits to produce average protein abundance values for PSI and PSII. The PSII to PSI ratio is the ratio of these average abundance values.

## Data Availability

The datasets presented in this manuscript are publically available at: https://massive.ucsd.edu/ProteoSAFe/datasets.jsp#%7B%22query%22%3A%7B%7D%2C%22table_sort_history%22%3A%22createdMillis_dsc%22%7D, dataset ID MSV000091808.
