# Supplementary material for: Protein assemblies in the Arabidopsis thaliana chloroplast compartment
Source: Front Plant Sci. 2024 Aug 16;15:1380969. doi: 10.3389/fpls.2024.1380969 (PMC11362043; doi:10.3389/fpls.2024.1380969)
Supplement: Supplementary file 1 [file Presentation1.pdf]

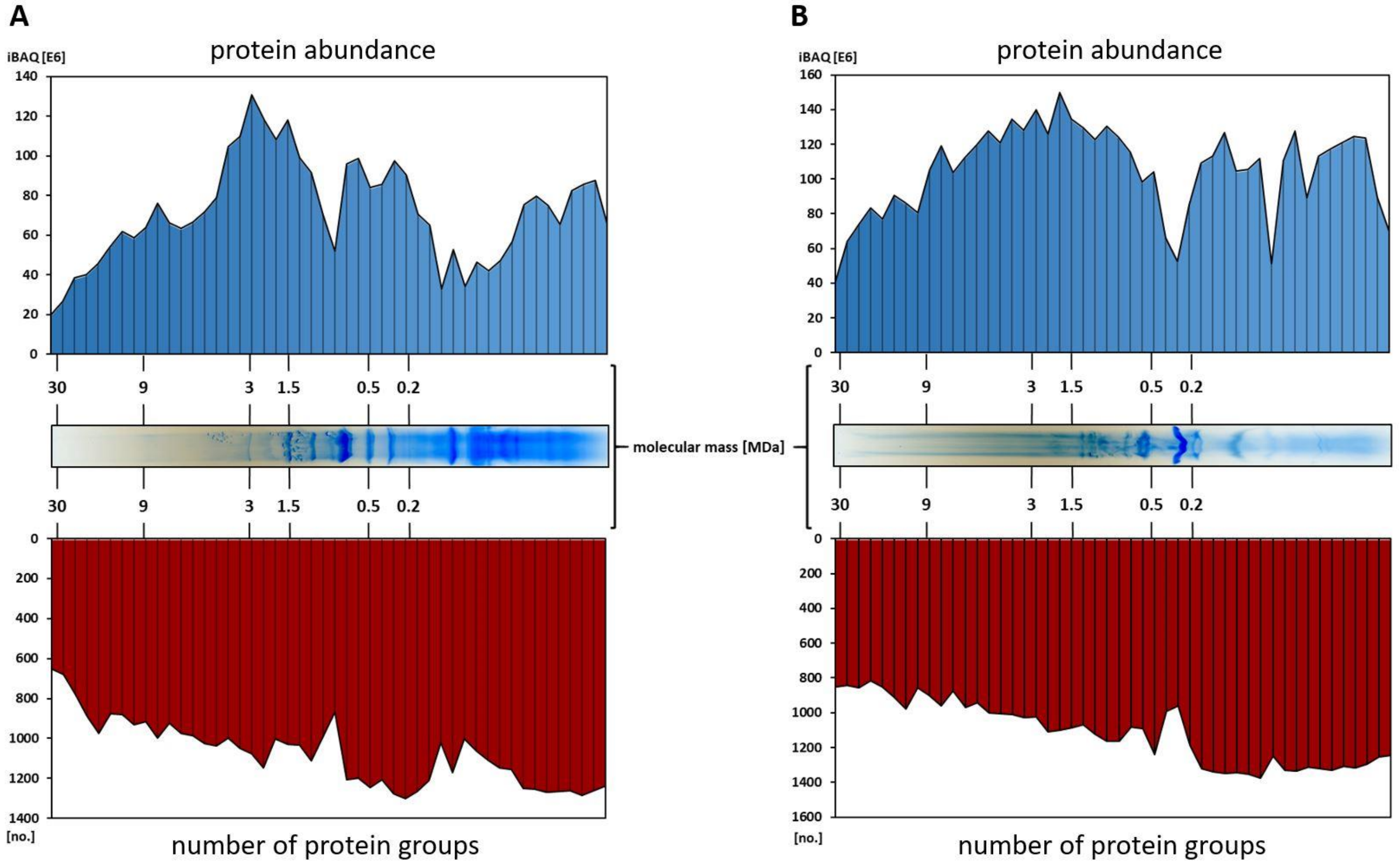

SUPPLEMENTARY FIGURE 1

Distribution of proteins across lpBN-PAGE fractions in the mitochondrial (A) and the chloroplast (B) isolates. Organelles were isolated in parallel and separated on the same lpBN-gel. Gel lanes were cut into 48 fractions each and subjected to the complexome profiling workflow by tryptic in-gel digestion for subsequent HPLC-timsTOF-MS analysis. Total protein abundance in each fraction was calculated by cumulating iBAQ values of all proteins identified in that fraction and is displayed in the blue graph. The number of identified protein groups in each fraction is given in the red graph at the bottom. Corresponding lpBN-PAGE lanes are displayed in the middle, with molecular masses indicated above and below in MDa. The molecular mass of each lpBN-PAGE fraction was calculated by the exponential interpolation algorithm of the NOVA software, using defined masses of respiratory protein complexes as well as the mitochondrial pyruvate dehydrogenase complex (see also Figures 2, 3).

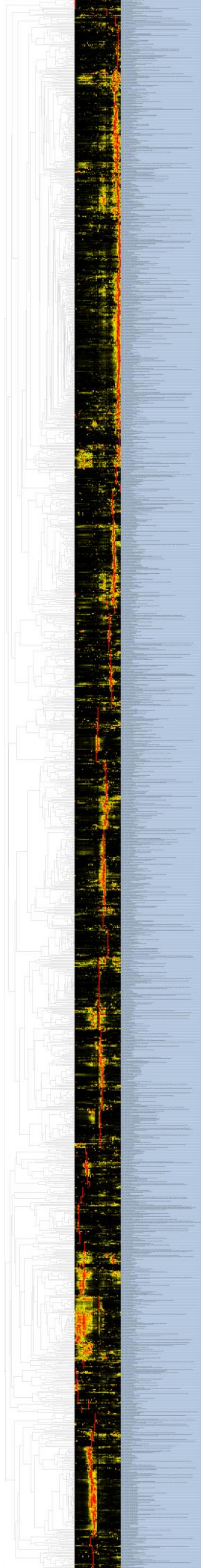

SUPPLEMENTARY FIGURE 2

Entire mitochondrial Complexome Map. The complete map in high resolution, including protein search function, can also be inspected under https://complexomemap.de/96.

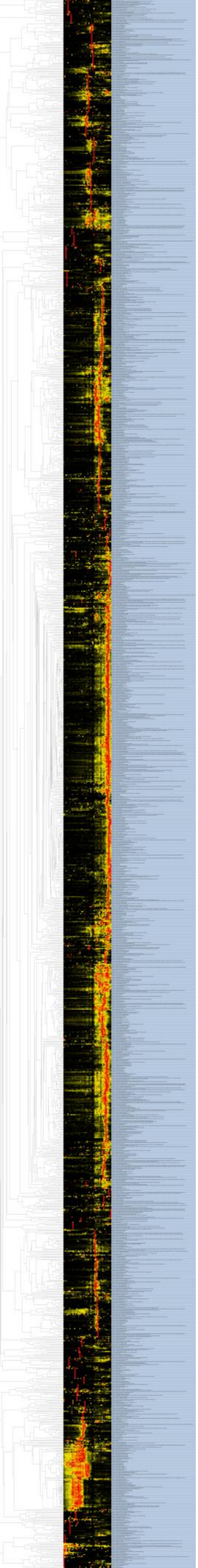

SUPPLEMENTARY FIGURE 3

Entire chloroplast Complexome Map. The complete map in high resolution, including protein search function, can also be inspected under https://complexomemap.de/95.

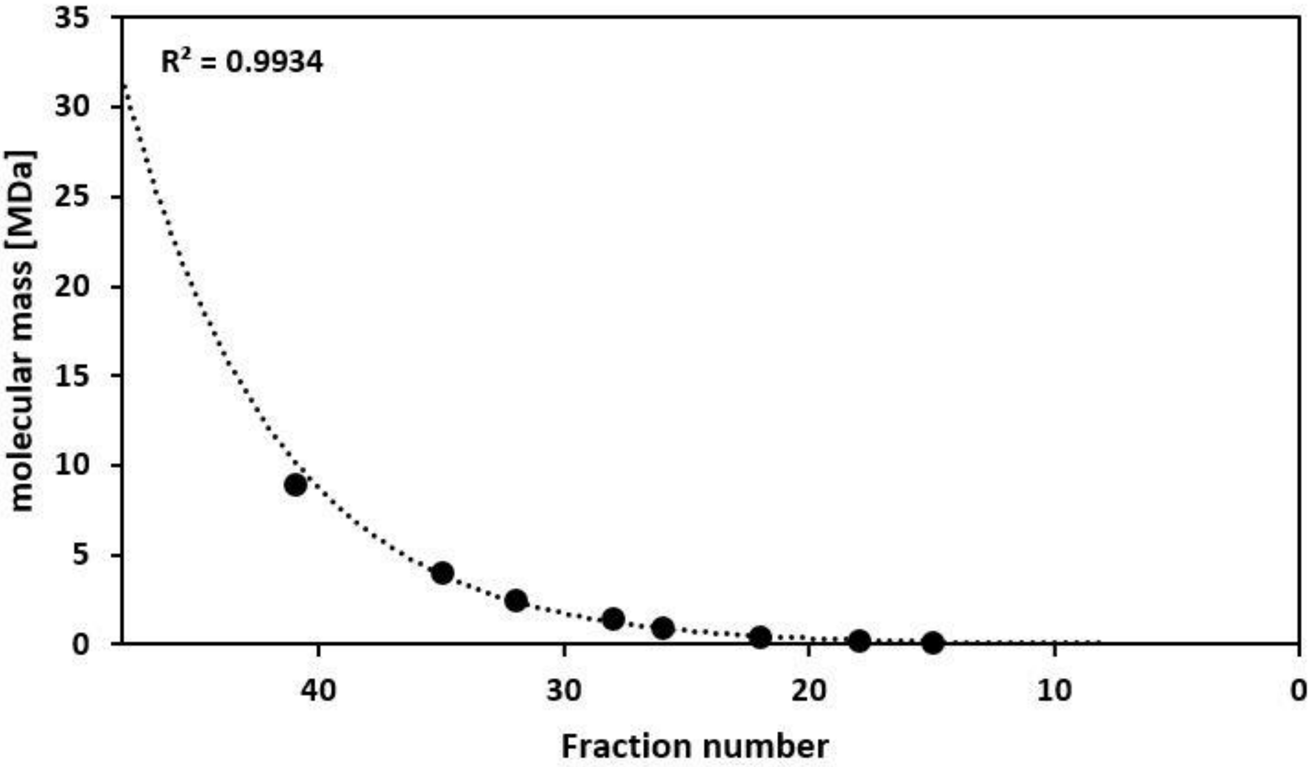

SUPPLEMENTARY FIGURE 4

Molecular mass calibration of lpBN-PAGE fractions using defined mitochondrial protein complexes as high molecular mass standards. Defined molecular masses of mitochondrial CII (0.16 MDa), CIV (0.21 MDa), Q4 CIII (0.5 MDa) and CI (1 MDa), as well as molecular masses of the CI+CIII 2 (1.5 MDa) and CI 2 +CIII 2 (2.5 MDa) super complexes were used. Additionally, the OGDC complex (4 MDa) and the PDC complex (9 MDa) served as high molecular mass standards. Molecular masses of adjacent fractions were calculated using the exponential interpolation algorithm of the NOVA software.

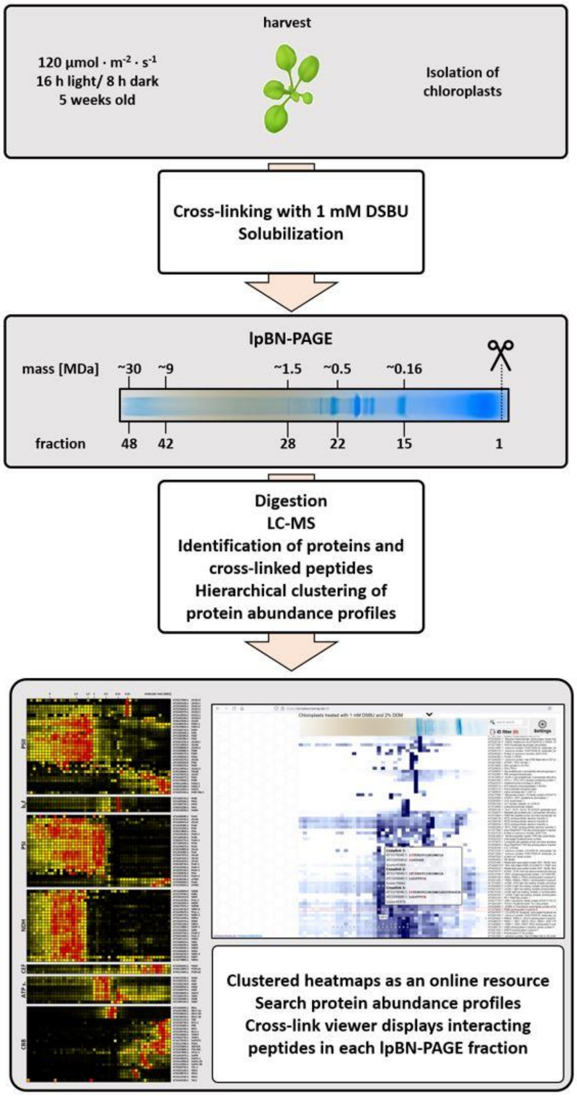

SUPPLEMENTARY FIGURE 5

CP workflow employing DSBU-cross-linking of isolated chloroplasts. Arabidopsis thaliana plants, ecotype Columbia-0, were grown under long day conditions (16 h light, 8 h darkness) using a photon flux density (PFD) of 120 µmol s -1 m -2 (first panel). After isolation, chloroplasts were cross-linked with 1 mM DSBU. Protein assemblies were solubilized with different detergents and separated according to their apparent molecular mass by lpBN-PAGE before stained gel lanes were sliced into 48 fractions (third panel). Each fraction was subjected to tryptic in-gel digestion before peptide mixtures of each fraction were analyzed by LC-timsTOF-MS (panel four). Recorded spectra were analyzed by MaxQuant to generate protein abundance profiles and to identify cross-linked peptides. Protein abundance profiles were then submitted to hierarchical clustering using the NOVA software. Resulting complexome maps were uploaded to https://complexomemap.de/projects-interaction-chloroplasts/, where interested readers can search for candidate proteins and potential interaction partners. Identified cross-links are marked by white, crossed-out circles. Hovering the pointer over individual coordinates triggers the appearance of a pop-up displaying additional information, such as peptide sequences, cross-link scores, and cross-linked amino acids (highlighted in red).

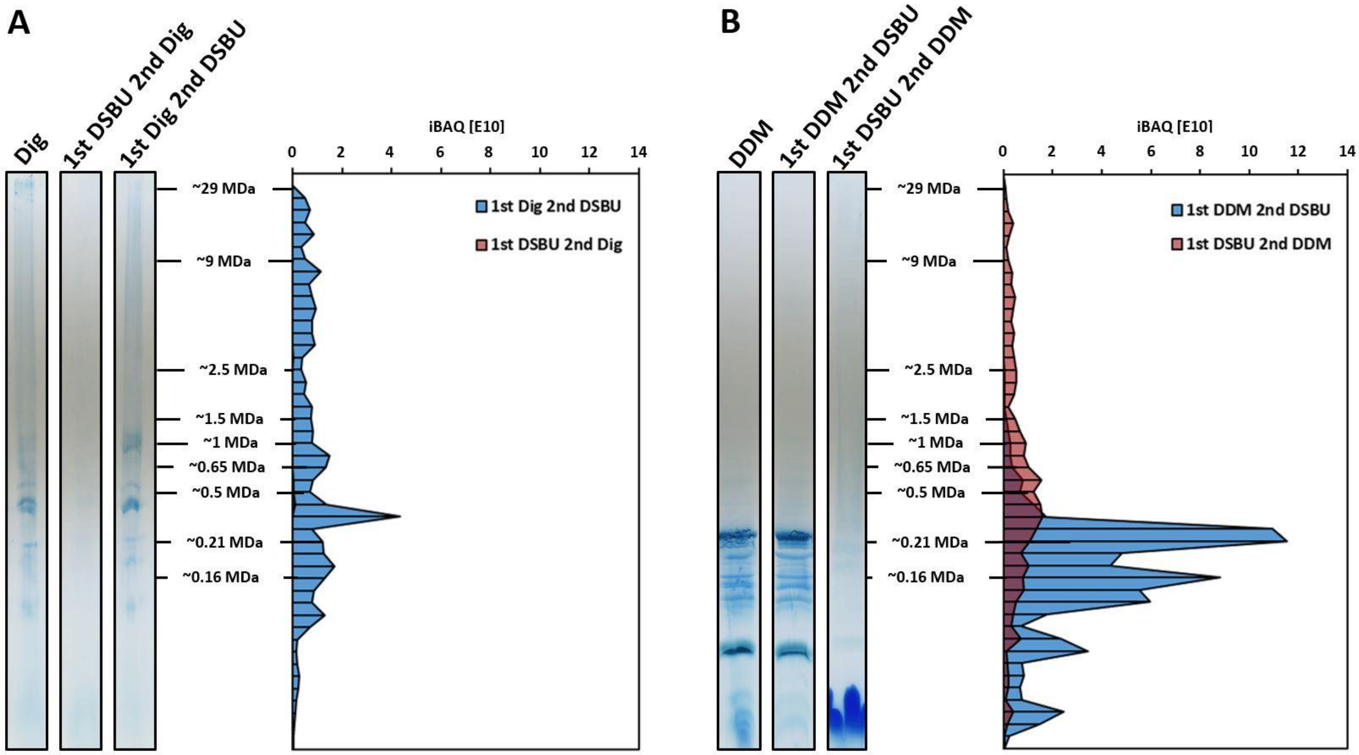

SUPPLEMENTARY FIGURE 6

Influence of the cross-linking strategy on the distribution of chloroplast protein abundance across lpBN-PAGE fractions. Freshly isolated chloroplasts were adjusted to a chlorophyll-concentration of 0.2 µg µl-1. Chloroplasts were either cross-linked with 5 mM DSBU before solubilization (1st DSBU 2nd Dig/DDM; red graphs), or solubilized with detergent before being submitted to cross-linking with 5 mM DSBU (1st Dig/DDM 2nd DSBU; blue graphs). As a control, chloroplasts were solubilized with detergent in the absence of any cross-linker. Samples were separated on the same lpBN gel. Protein abundance in each lpBN-PAGE fraction is based on cumulated iBAQ values of all proteins identified within that fraction. (A), solubilization using 2.5% [w/v] digitonin; (B), solubilization using 2% [w/v] DDM.

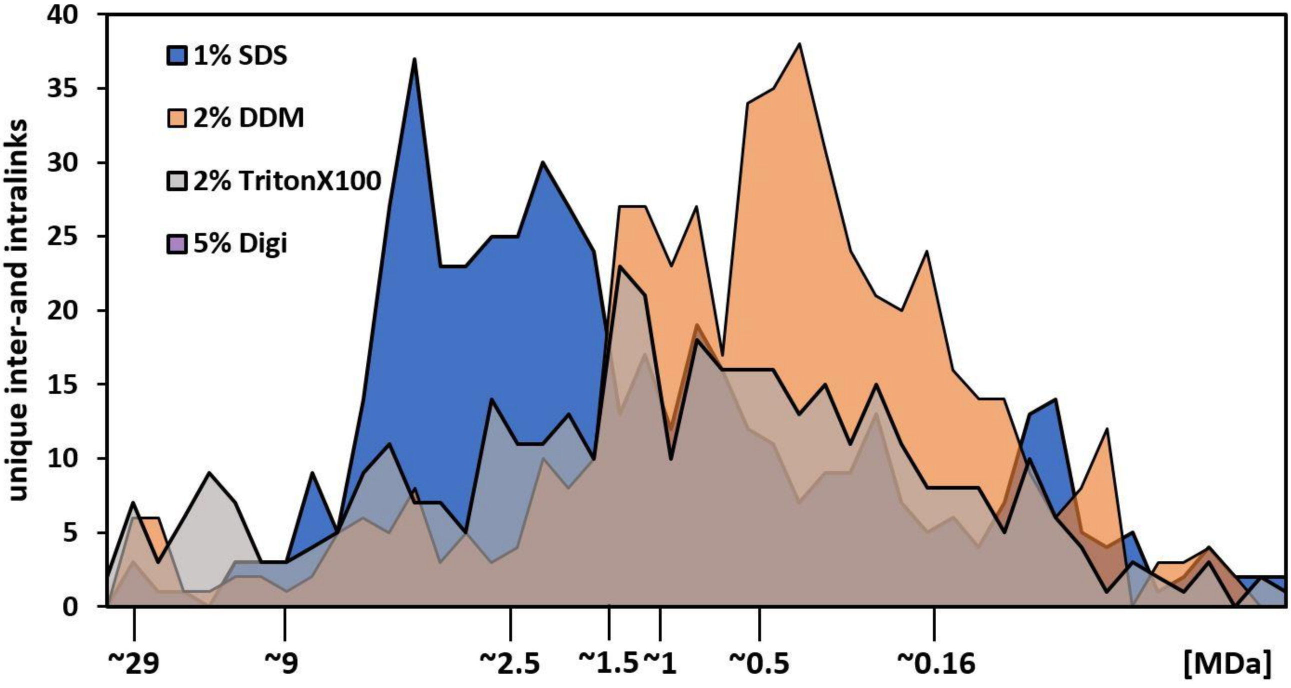

SUPPLEMENTARY FIGURE 7

Influence of different detergents on the detection of intralinks and interlinks in lpBN fractions. Freshly isolated chloroplasts were treated with 1 mM DSBU before detergent solubilization with 5% [w/v] digitonin (purple), 2% [w/v] DDM (orange), 2% [v/v] Triton X-100 (grey) and 1% [w/v] SDS (blue). Chloroplast protein equivalent to 20 µg chlorophyll was loaded on each lane. Intralinks and interlinks identified by MaxQuant in each fraction of the four complexomes presented were cumulated.

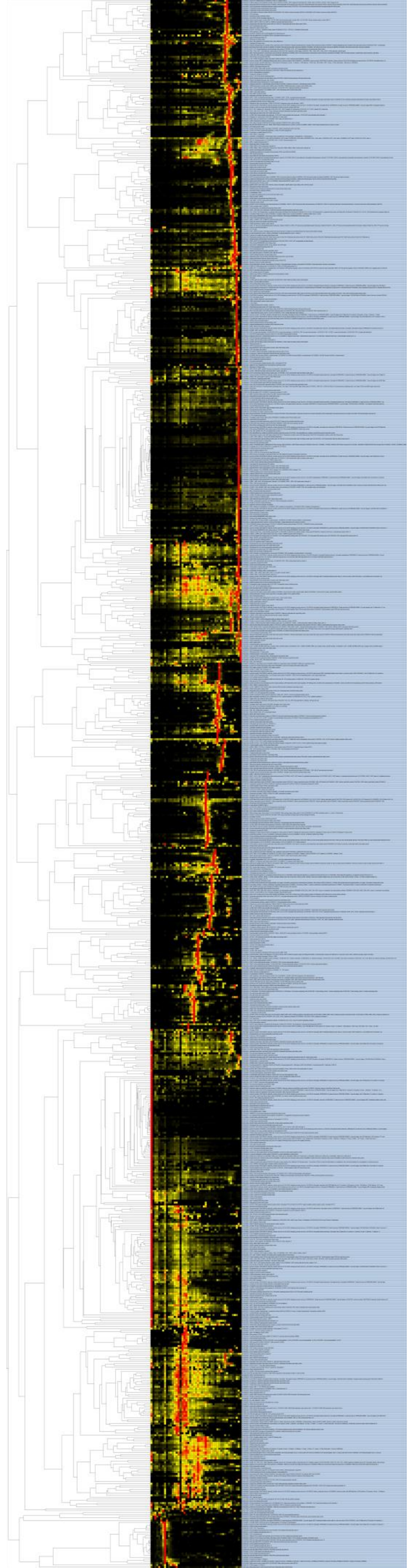

SUPPLEMENTARY FIGURE 8

Complexome map obtained from chloroplast proteins after cross-linking with 1 mM DSBU and SDS-solubilization.

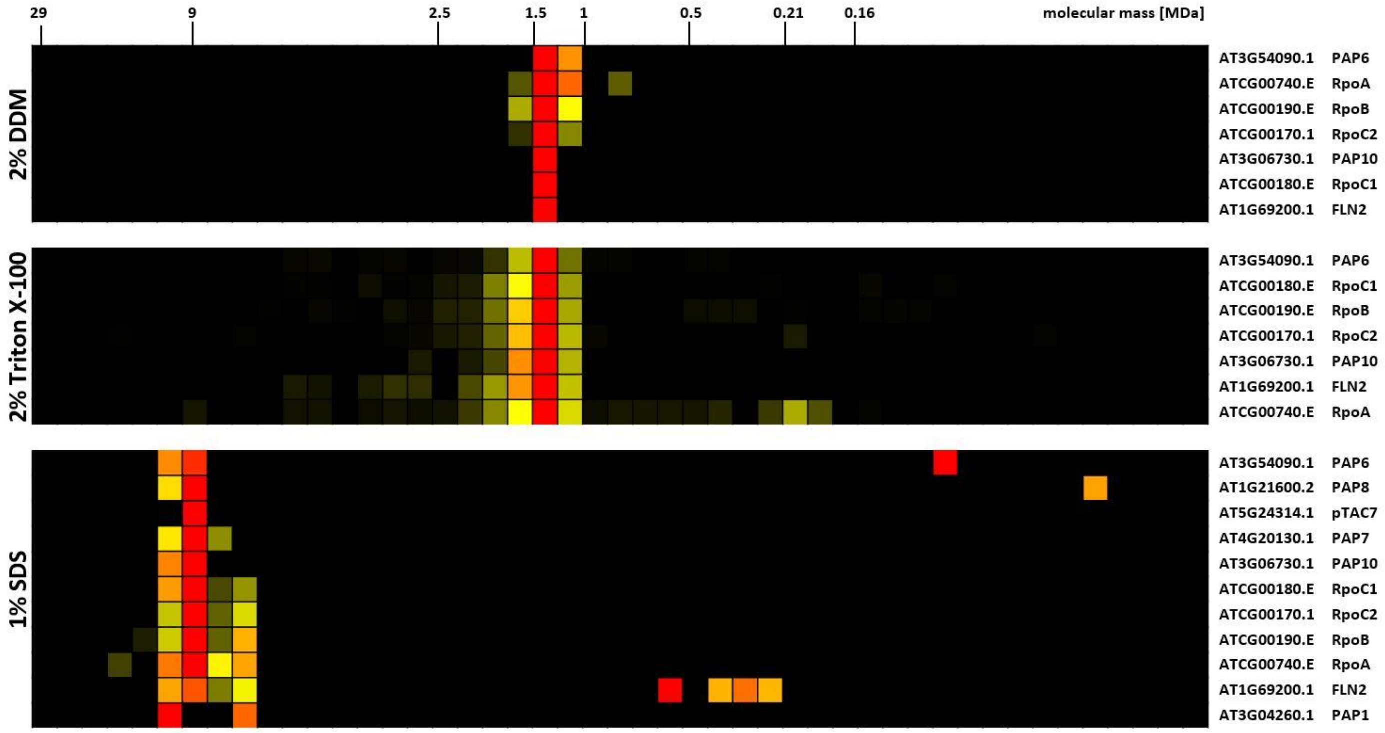

SUPPLEMENTARY FIGURE 9

Abundance profiles of PEP subunits cross-linked with 1 mM DSBU before solubilization with different detergents. PPIs were stabilized after chloroplast isolation by cross-linking with 1 mM DSBU and protein assemblies were subsequently solubilized with 2% DDM, 2% Triton X-100 or 1% SDS, and subsequently separated by lpBN PAGE. Resulting gel lanes were fractionated and subjected to tryptic in-gel digestion before each fraction was analyzed by LC-tims-TOF-MS. Resulting abundance profiles of PEP subunits were clustered hierarchically. Detergent treatments are indicated to the left, Arabidopsis gene identifier and protein names are given to the right. Molecular masses are given on top in MDa. High protein abundance is indicated in red, medium protein abundance in orange, low protein abundance is depicted in yellow, whereas protein abundance below detection limit is displayed in black

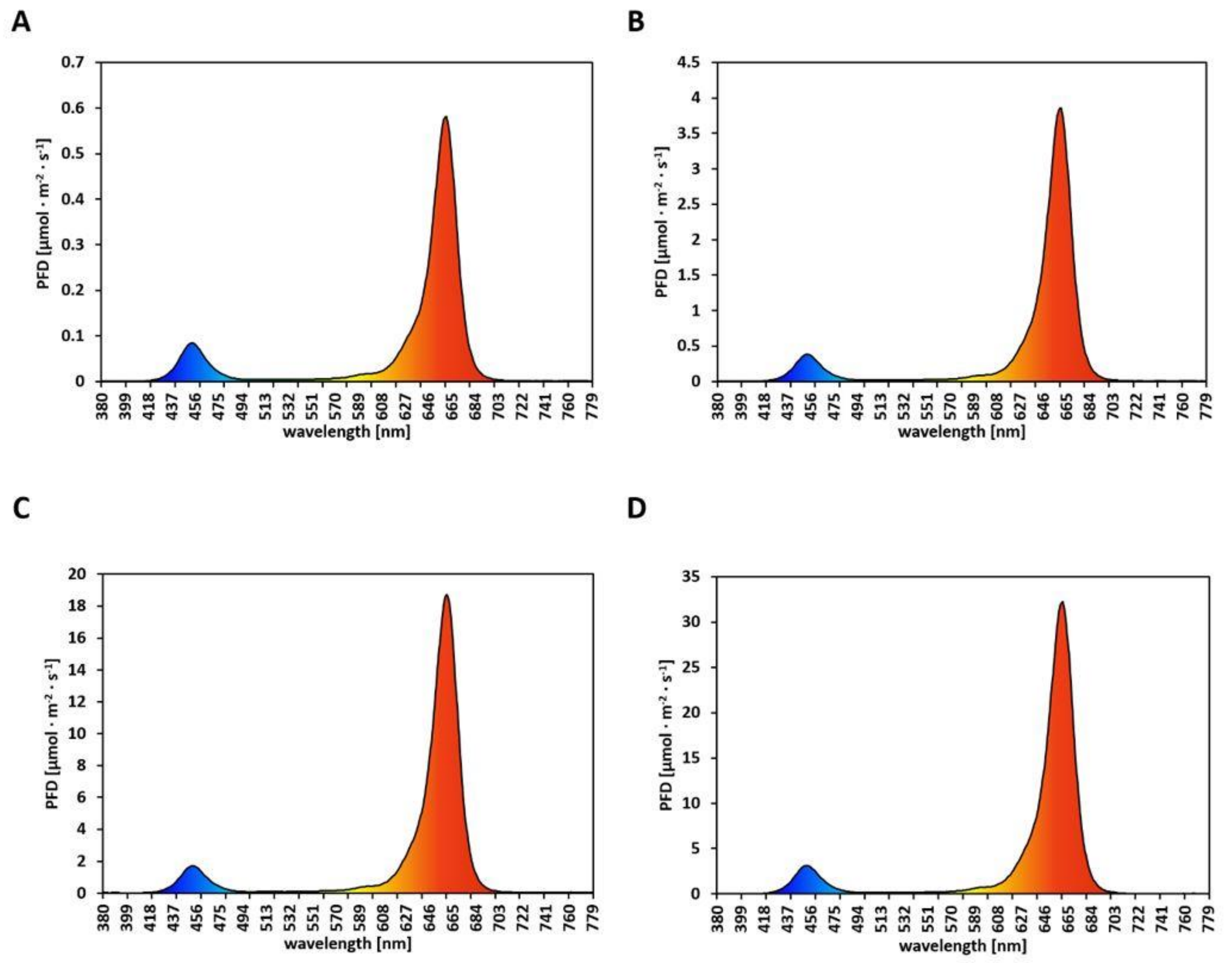

SUPPLEMENTARY FIGURE 10

Light spectra of the 300 W LED lamp at different PFD values. The PFD was adjusted to (A) 20, (B) 120, (C) 600 and (D) 1000 µmol photons · m -2 · s -1 by altering the distance between light source and plants. Each graph shows the PFD in relation to the respective wavelength in nanometer (nm) at the level of the plants. Plants were positioned under the LED clusters during the dark period and were then kept under the particular PFDs for 8 h before harvest.

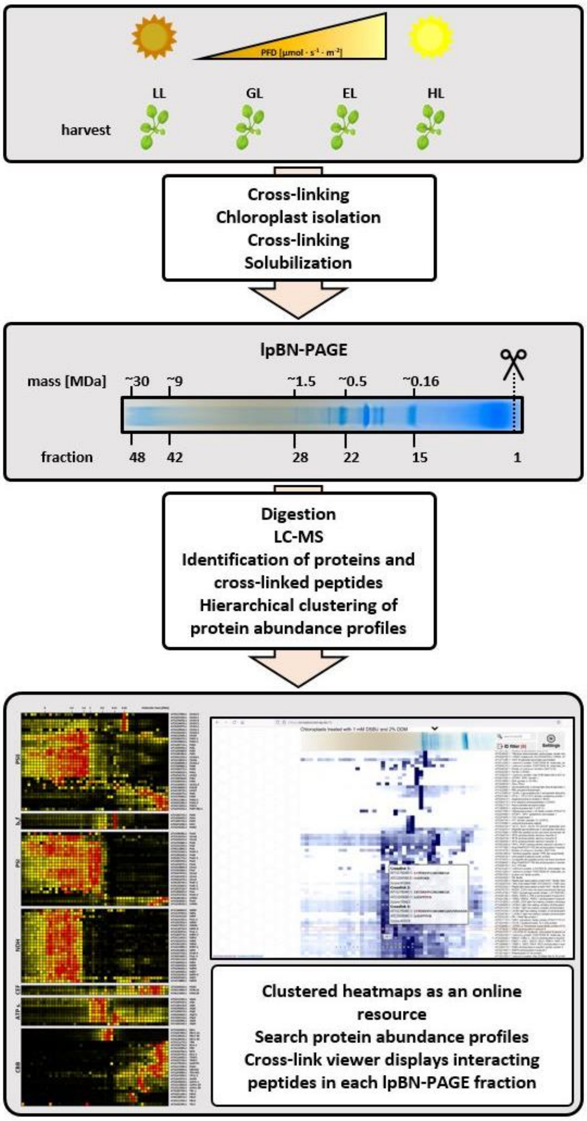

SUPPLEMENTARY FIGURE 11

CP workflow employing DSBU-cross-linking of the raw extract and again of isolated chloroplasts. Arabidopsis thaliana plants, ecotype Columbia-0, were grown under long day conditions (16 h light, 8 h darkness) using a photon flux density (PFD) of 120 µmol s -1 m -2 (first panel). Directly after cell disruption and organelle isolation, chloroplasts were cross-linked with 1 mM DSBU before being solubilized with different detergents. Solubilized protein complexes were separated according to their apparent molecular mass by lpBN-PAGE before stained gel lanes were sliced into 48 fractions (third panel). Each fraction was subjected to tryptic in-gel digestion before peptide mixtures of each fraction were analyzed by LC-timsTOF-MS (panel four). Recorded spectra were analyzed by MaxQuant to generate protein abundance profiles and to identify cross-linked peptides. Complexome maps were uploaded to https://complexomemap.de/projects-interaction.chloroplasts. Interested readers can search for candidate proteins and potential interaction partners. Identified cross-links are marked by white, crossed-out circles. Hovering the pointer over individual coordinates triggers the appearance of a pop-up displaying additional information, such as peptide sequences, cross-link scores, and cross-linked amino acids (highlighted in red).

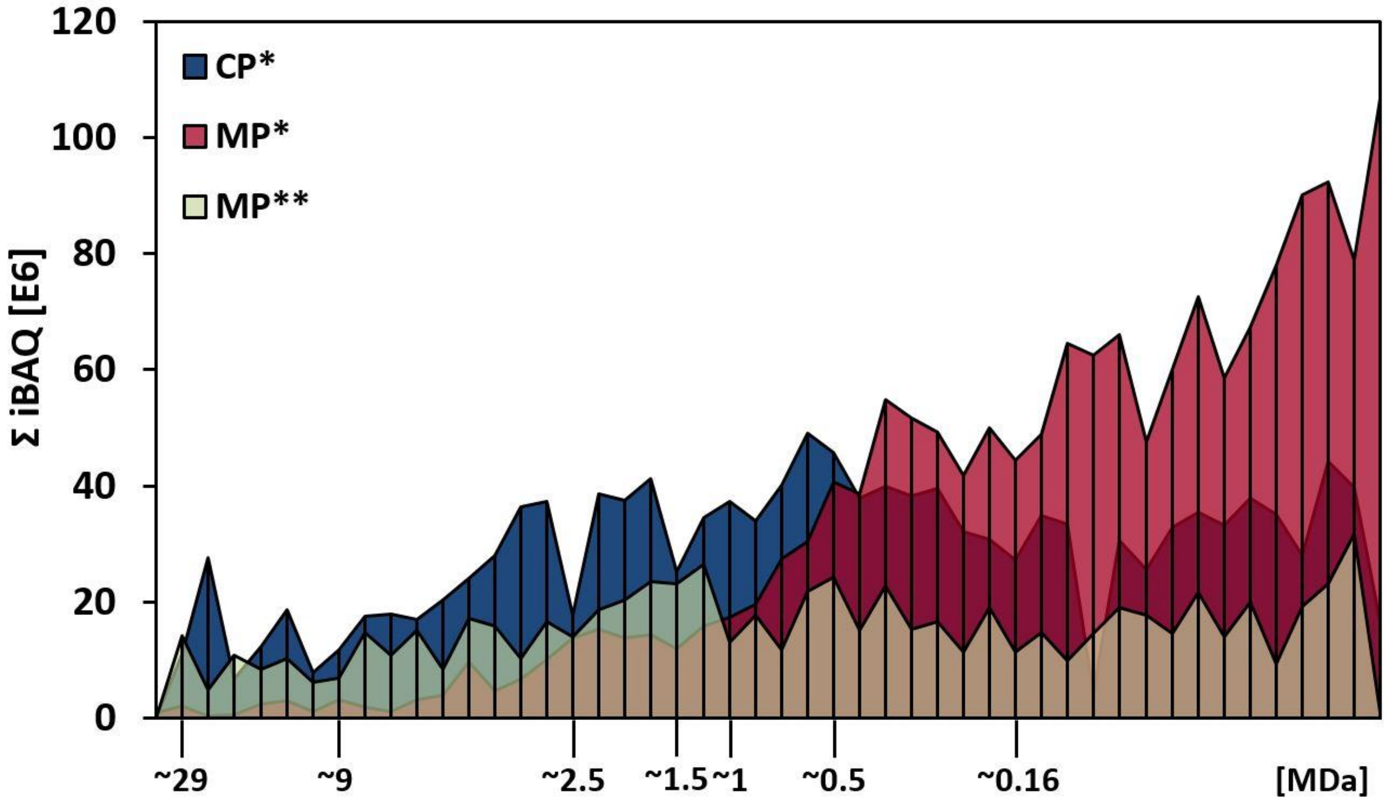

SUPPLEMENTARY FIGURE 12

Influence of the chloroplast isolation protocol on the distribution of protein abundance across lpBN-gel fractions. Chloroplasts were either isolated according to the conventional isolation procedure followed by a single DSBU cross-linking (classic preparation, CP*; blue graph), or according to an isolation protocol using only a fraction of the starting material, thus allowing DSBU cross-linking directly after cell disruption (mini-preparation, MP*; red graph). Additionally, chloroplasts isolated following the MP* protocol were cross-linked a second time after chloroplast purification (MP**, green graph).

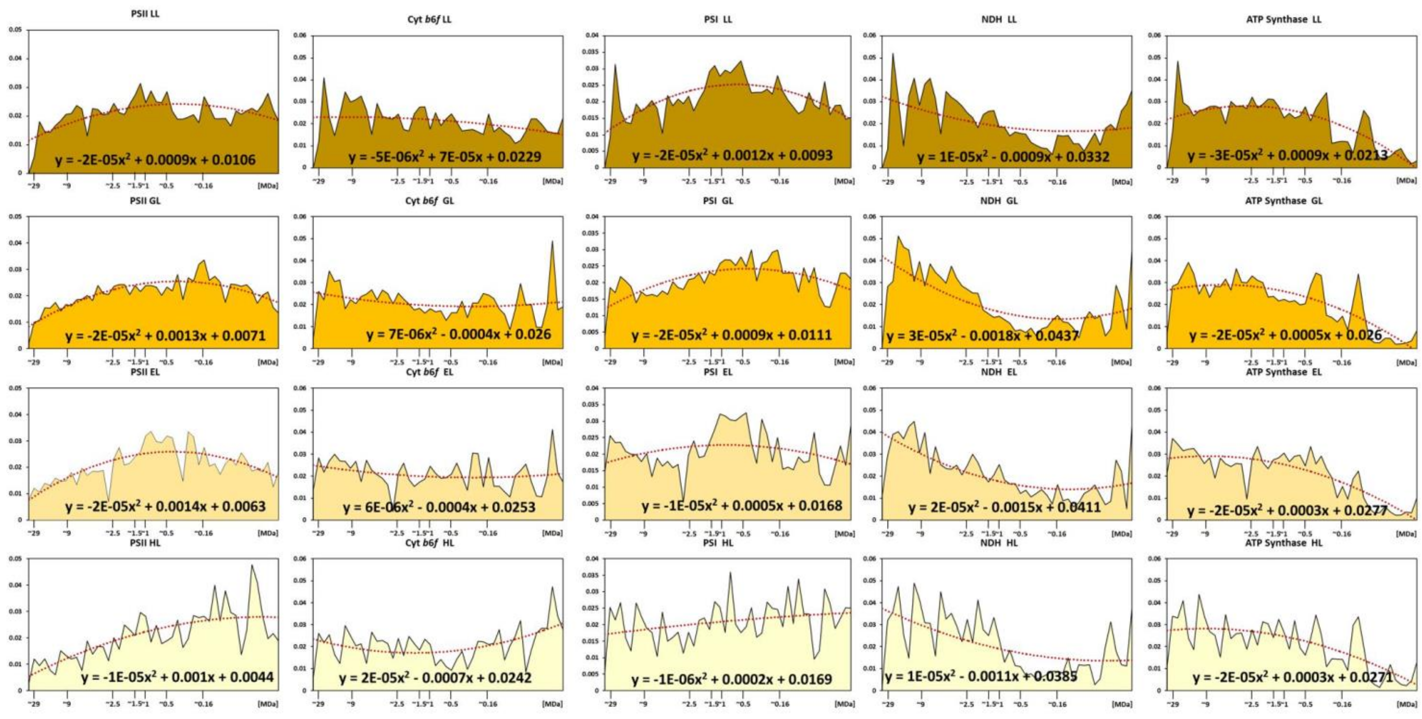

SUPPLEMENTARY FIGURE 13

Distribution of normalized thylakoid protein complex abundance isolated from plants acclimated to increasing light intensities. Protein complex abundance was computed by cumulating iBAQ values from all identified subunits of a complex for each individual fraction. Cumulated abundance in each fraction was normalized to the total abundance of the complex in the complexome (summed up iBAQ values of all subunits and all fractions). Abundance distributions of complexes acclimated to low light (LL) are displayed in brown, while distributions of complexes acclimated to growth light (GL) are depicted in orange, enhanced light (EL) acclimated complex distributions are shown in bright yellow, high light (HL) acclimated abundance distributions are shown in whitish. For each normalized abundance profile, a polynomial fit was calculated, which is displayed as a red-dotted line. Formulas of the polynomial fits are given at the bottom of each diagram.

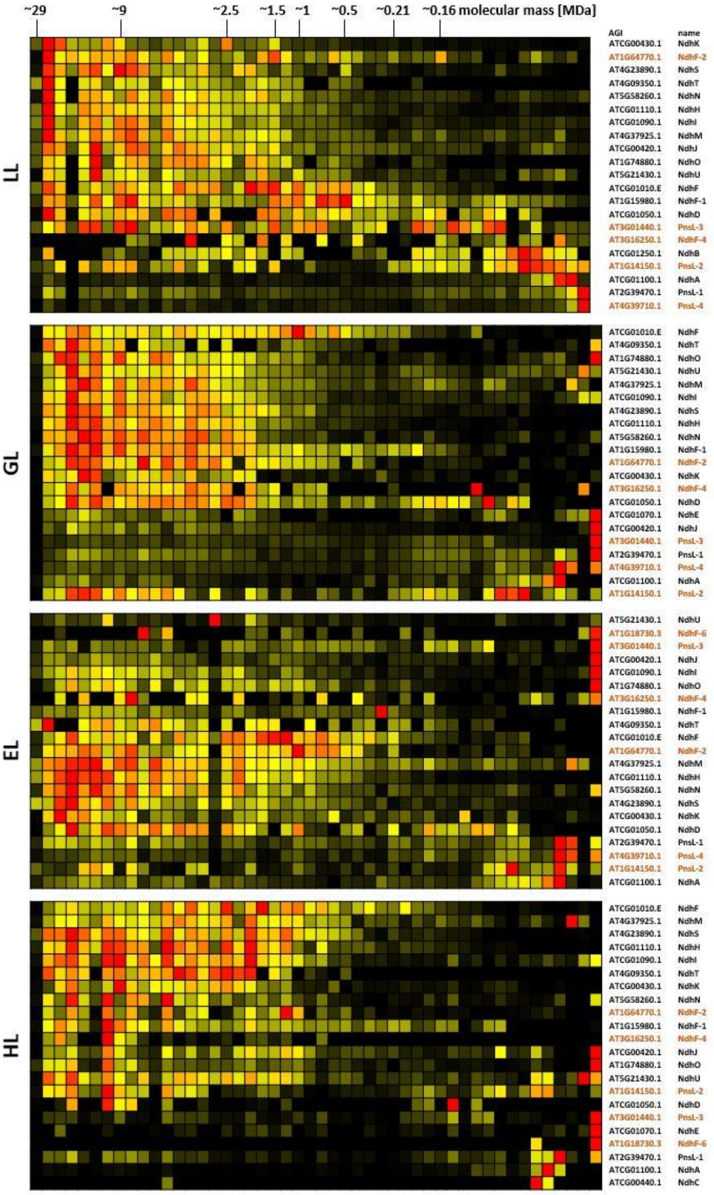

SUPPLEMENTARY FIGURE 14

Complexome maps displaying abundance profiles of NDH subunits of leaves acclimated to different light intensities. Plants grown under increasing light intensities (low light, “LL”, 20 µmol · m-2 · s-1 ; grow light, “GL”, 120 µmol · m-2 · s-1; enhanced light, “EL”, 600 µmol · m-2 · s-1; high light, “HL”, 1000 µmol · m-2 · s-1 were harvested) in parallel. PPIs were stabilized before and after chloroplast isolation by DSBU cross-linking, subsequently solubilized with 1% SDS and subjected to the CP workflow shown in Supplementary Figure 11. Protein assemblies of all treatments were separated on the same lpBN-gel to reduce technical variations. Abundance profiles of NDH subunits were manually selected and subsequently hierarchically clustered by NOVA. The light treatment is indicated to the left, Arabidopsis gene identifier and protein names are given to the right (isoforms written in brown). Molecular masses of complexome fractions are given on top in MDa. High normalized protein abundance is indicated in red, medium protein abundance in orange, low protein abundance is depicted in yellow, whereas no detectable protein abundance is displayed in black.

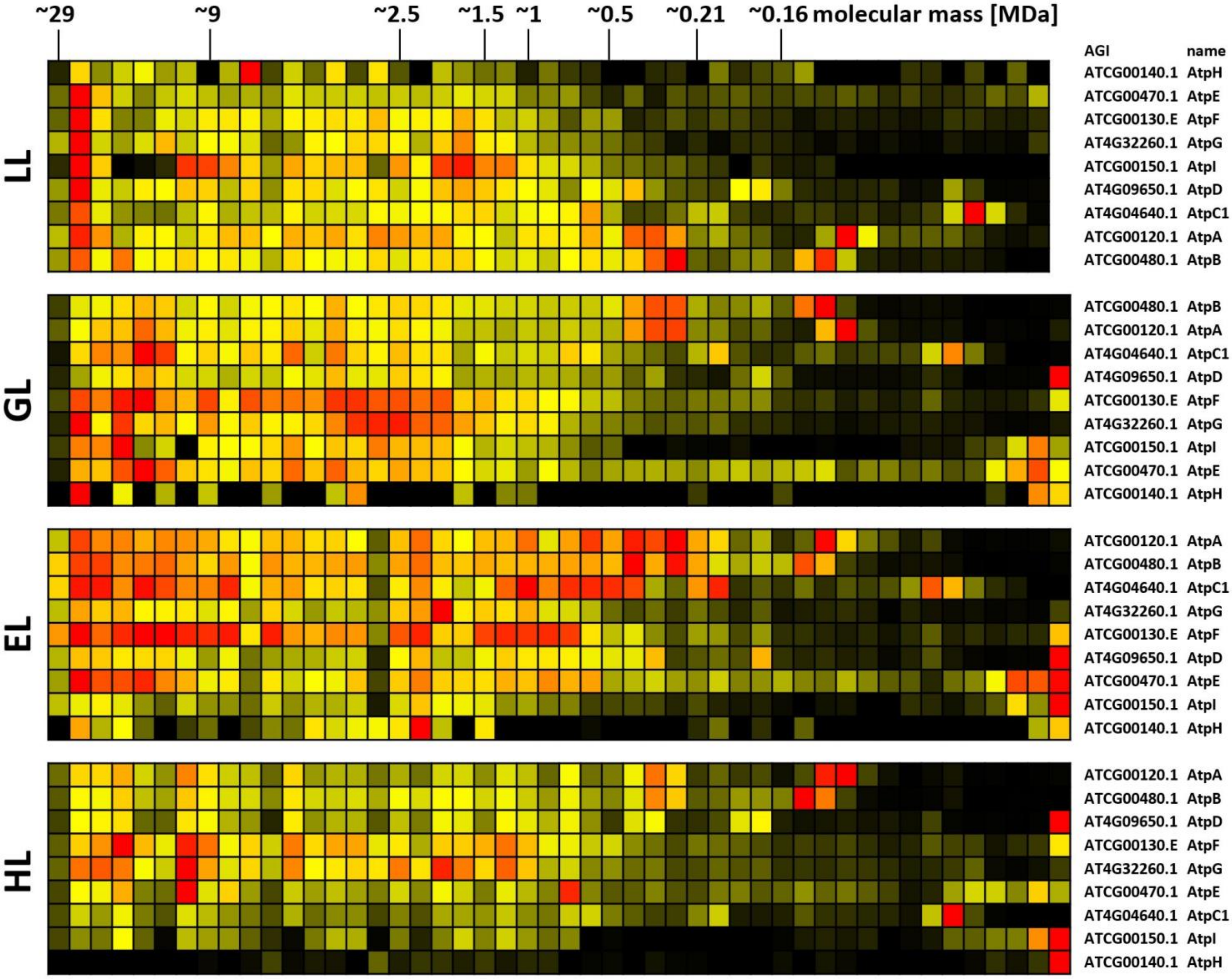

SUPPLEMENTARY FIGURE 15

Protein abundance profiles of ATP-synthase subunits of leaves after acclimation to different light intensities. Plants grown under increasing light intensities (low light, “LL”, 20 µmol · m -2 · s -1 ; grow light, “GL”, 120 µmol · m -2 · s -1; enhanced light, “EL”, 600 µmol · m -2 · s -1; high light, “HL”, 1000 µmol · m -2 · s -1) were harvested in parallel. PPIs were stabilized before and after chloroplast isolation by cross-linking with 1 mM DSBU. Protein assemblies were subsequently solubilized with 1% [w/v] SDS and subjected to the CP workflow shown in Supplementary Figure 11. Abundance profiles of ATP-synthase subunits were manually selected and subsequently hierarchically clustered by NOVA. The light treatment is indicated to the left, Arabidopsis gene identifier and protein names are given to the right (isoforms are written in brown). Molecular masses of complexome fractions are given on top in MDa. High protein abundance is indicated in red, medium protein abundance in orange, low protein abundance is depicted in yellow, whereas non-detectable protein abundance is displayed in black. Protein assemblies of all treatments were separated on the same lpBN-gel to reduce technical variation.

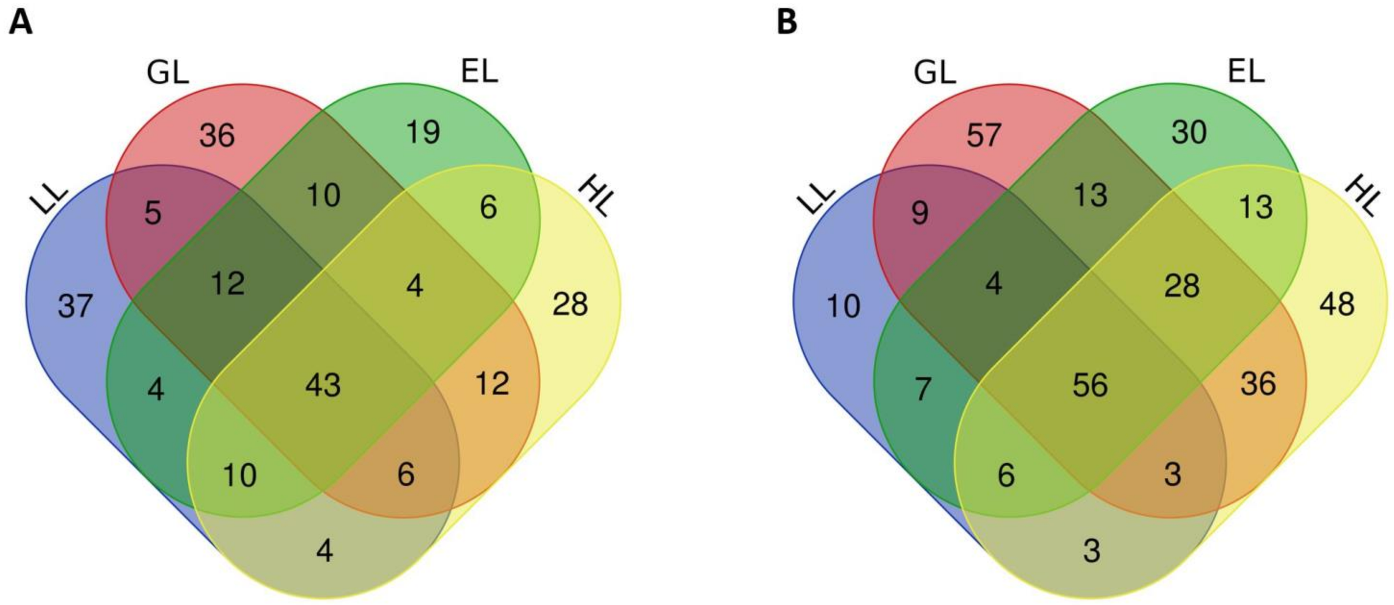

SUPPLEMENTARY FIGURE 16

Distribution of unique cross-linking products within the different datasets. Non-redundant inter-and intra-cross-links identified across all fractions of the complexome maps acclimated to low light (LL), growth light (GL), enhanced light (EL) or high light (HL) were compared against each other. (A), inter-links; (B), intra-links.

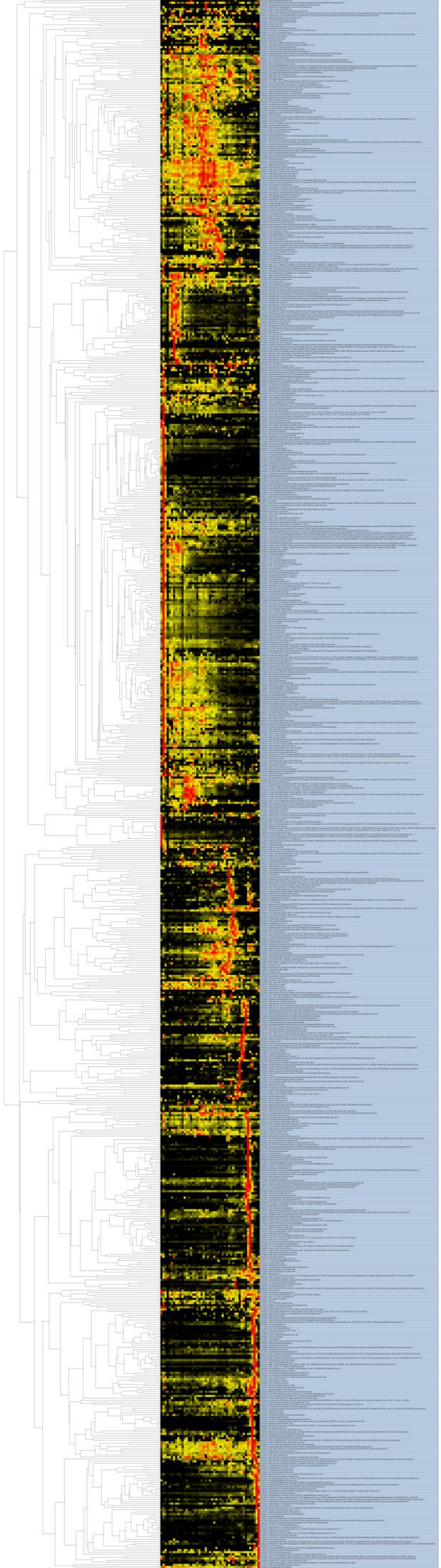

SUPPLEMENTARY FIGURE 17

Complexome map obtained from chloroplast isolated from plants exposed to low light (20 µmol · m^{-2} · s^{-1}) conditions. The complete map can also be inspected under https://complexomemap.de/112.

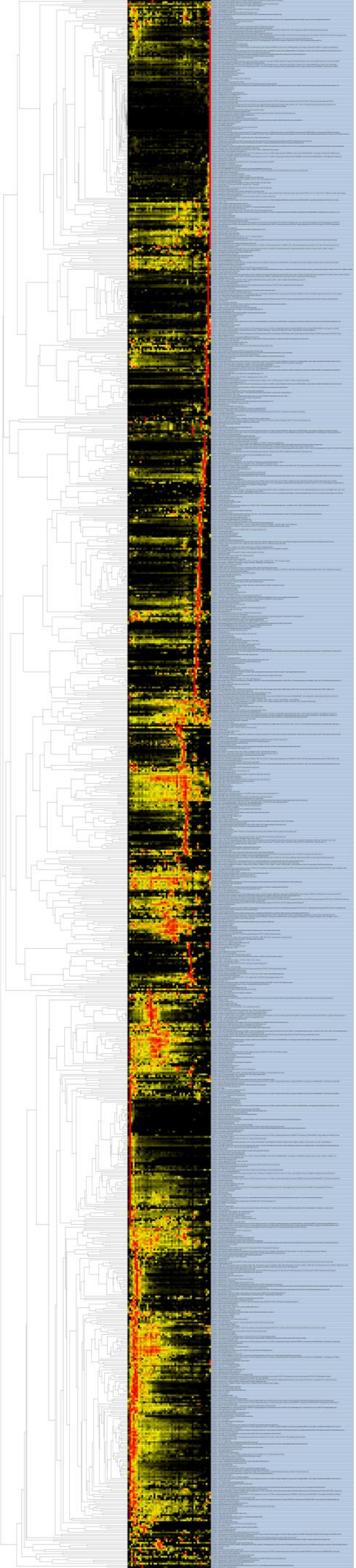

SUPPLEMENTARY FIGURE 18

Complexome map obtained from chloroplast isolated from plants exposed to growth light (120 µmol · m^{-2} · s^{-1}) conditions. The complete map can also be inspected under https://complexomemap.de/111.

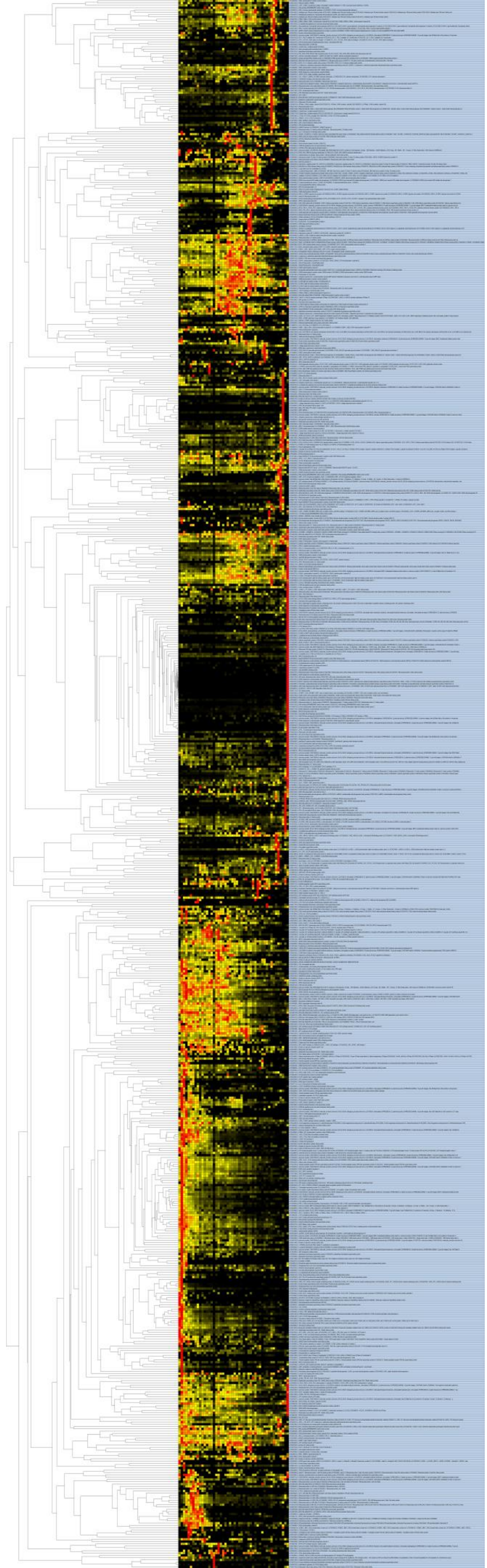

SUPPLEMENTARY FIGURE 19

Complexome map obtained from chloroplast isolated from plants exposed to enhanced light (600 µmol · m^{-2} · s^{-1}) conditions. The complete map can also be inspected under https://complexomemap.de/113.

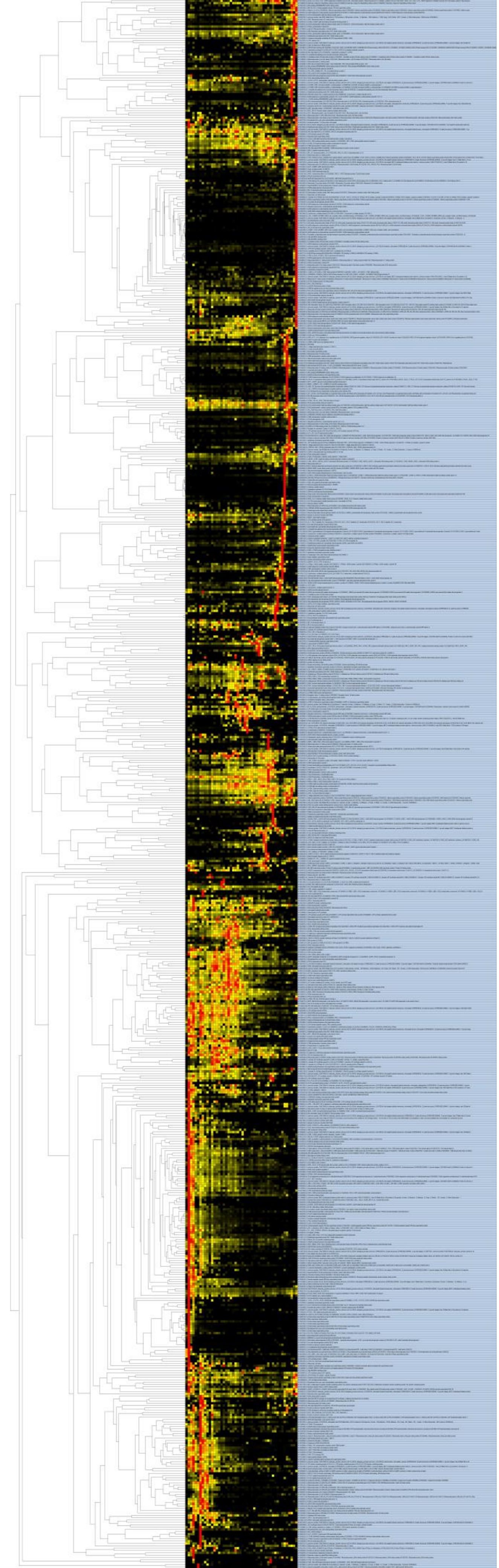

SUPPLEMENTARY FIGURE 20

Complexome map obtained from chloroplast isolated from plants exposed to high light (1000 µmol · m^{-2} · s^{-1}) conditions. The complete map can also be inspected under https://complexomemap.de/114.
